# Subtyping *Cryptosporidium ubiquitum,*a Zoonotic Pathogen Emerging in Humans

**DOI:** 10.3201/eid2002.121797

**Published:** 2014-02

**Authors:** Na Li, Lihua Xiao, Keri Alderisio, Kristin Elwin, Elizabeth Cebelinski, Rachel Chalmers, Monica Santin, Ronald Fayer, Martin Kvac, Una Ryan, Bohumil Sak, Michal Stanko, Yaqiong Guo, Lin Wang, Longxian Zhang, Jinzhong Cai, Dawn Roellig, Yaoyu Feng

**Affiliations:** East China University of Science and Technology, Shanghai, China (N. Li, Y. Guo, L. Wang, Y. Feng);; Centers for Disease Control and Prevention, Atlanta, Georgia, USA (N. Li, L. Xiao, Y. Guo, D. Roellig);; New York City Department of Environmental Protection, Flushing, New York, USA (K. Alderisio);; UK *Cryptosporidium* Reference Unit, Swansea, UK (K. Elwin, R. Chalmers);; Minnesota Department of Health, St. Paul, Minnesota, USA (E. Cebelinski);; US Department of Agriculture, Beltsville, Maryland, USA (M. Santin, R. Fayer);; Academy of Science of Czech Republic, České Budějovice, Czech Republic (M. Kvac, B. Sak);; Murdoch University, Perth, Australia (U. Ryan);; Slovak Academy of Sciences, Košice, Slovakia (M. Stanko);; Henan Agricultural University, Zhengzhou, China (L. Zhang);; Qinghai Academy of Veterinary Medicine and Animal Science, Xining, China (J. Cai)

**Keywords:** Cryptosporidiosis, *Cryptosporidium*, zoonoses, epidemiology, molecular typing,whole genome sequencing, genomics, ruminants, rodents, horse, raccoon, sifaka, parasites, humans

## Abstract

*Cryptosporidium ubiquitum* is an emerging zoonotic pathogen. In the past, it was not possible to identify an association between cases of human and animal infection. We conducted a genomic survey of the species, developed a subtyping tool targeting the 60-kDa glycoprotein (gp60) gene, and identified 6 subtype families (XIIa–XIIf) of *C. ubiquitum*. Host adaptation was apparent at the gp60 locus; subtype XIIa was found in ruminants worldwide, subtype families XIIb–XIId were found in rodents in the United States, and XIIe and XIIf were found in rodents in the Slovak Republic. Humans in the United States were infected with isolates of subtypes XIIb–XIId, whereas those in other areas were infected primarily with subtype XIIa isolates. In addition, subtype families XIIb and XIId were detected in drinking source water in the United States. Contact with *C. ubiquitum*–infected sheep and drinking water contaminated by infected wildlife could be sources of human infections.

*Cryptosporidium* infection is a leading cause of diarrhea in humans (*1*). Five *Cryptosporidium* species—*C. hominis*, *C. parvum*, *C. meleagridis*, *C. felis*, and *C. canis—*are responsible for most cases of cryptosporidiosis in humans. Among them, *C. hominis* and *C. parvum* are the most common etiologic agents, and the latter is responsible for most zoonotic infections (*2*). In recent years, *C. ubiquitum,* previously known as the cervine genotype, has been emerging as another major zoonotic species that infects persons. It has been found in humans worldwide, primarily in industrialized nations (*3*–*11*). In the United Kingdom, more human cases of cryptosporidiosis have been attributed to *C. ubiquitum* than to *C. canis* (*9*).

*C. ubiquitum* is of public health concern because of its wide geographic distribution and broad host range. Of all *Cryptosporidium* spp. identified by molecular diagnostic tools, it infects the greatest variety of host species (*12*). *C. ubiquitum* has been commonly detected in domestic and wild ruminants (sheep, goats, mouflon sheep, blesboks, nyalas, white-tailed deer, Père David’s deer, sika deer, ibexes, buffalos, and yaks), rodents (squirrels, chipmunks, woodchucks, beavers, porcupines, deer mice, house mice, and gerbils), carnivores (raccoons), and primates (lemurs and humans) (*12*–*16*). It has also been found in drinking source water, storm water runoff, stream sediment, and wastewater in various geographic locations (*17*–*22*).

Thus far, showing an association between human and animal cases of *C. ubiquitum* infection has not been possible because of the lack of suitable genetic markers for subtyping. For *C. parvum*, *C. hominis*, and some genetically related species, the most commonly used marker for subtyping is the 60-kDa glycoprotein gene (gp60, also called gp40/15). Sequence analysis of the gp60 gene has been used in studies of the genetic diversity, host adaptation, infection sources, and transmission dynamics of these *Cryptosporidium* spp. (*2*). However, it has been suggested that a single locus, such as gp60, is not a reliable marker of *C. parvum* and *C. hominis* population structure because genetic recombination may occur (*23*).

Because *C. ubiquitum* is genetically distant from *C. hominis* and *C. parvum*, its homologue of the gp60 gene has thus far not been identified (*24*). In this study, we identified the gp60 gene of *C. ubiquitum* by whole-genome sequencing and used it to develop a subtyping technique to characterize specimens from humans, various animals, and drinking source water.

## Materials and Methods

### Specimens

DNA extracts from 188 *C. ubiquitum*–positive specimens (collected during 2002–2012) were used, including those from animals, humans, and drinking source water. Animal specimens were obtained from various species of ruminants and rodents, a horse, a raccoon, and a primate, Verreaux’s sifaka (*Propithecus verreauxicoquereli*). Animal specimens were collected in the United States, Peru, Brazil, the United Kingdom, Spain, the Czech Republic, the Slovak Republic, Turkey, Algeria, South Africa, China, Nepal, and Australia (Table 1). Human specimens were obtained from the United States, Canada, Peru, the United Kingdom, and Turkey (Table 1). Water samples were collected from storm water and river water in the United States. The storm water samples were collected from a drinking-source watershed in New York where most of the rodent specimens were also collected (*13*,*17*). These specimens were initially found to be positive for *C. ubiquitum* by DNA sequence analysis of an ≈830-bp fragment of the small-subunit rRNA gene (*25*).

**Table 1:** Origin and gp60 subtype identity of *Cryptosporidium ubiquitum* specimens from human, animals, and water, 2002–2012

Host group/species	Total no. samples	Source location (no. samples)	Subtype family (no. samples)
Rodent			
Eastern chipmunk	2	USA (2)	XIIb (1), XIId (1)
Eastern gray squirrel	2	USA (2)	XIId (2)
Red squirrel	1	USA (1)	XIId (1)
Woodchuck	1	USA (1)	XIId (1)
Beaver	1	USA (1)	XIId (1)
Ring-tailed porcupine	2	USA (2)	XIIc (2)
Goat through porcupine*	3	USA (3)	XIIc (3)
Yellow-necked field mouse	2	Slovak Republic (2)	XIIe (1), XIIf (1)
Striped field mouse		Slovak Republic (2)	XIIe (2)
Carnivore			
Raccoon	1	USA (1)	XIId (1)
Parissodactyla			
Horse	1	UK (1)	XIIa (1)
Ruminant			
Sheep	56	USA (14), Peru (1), Brazil (3), Spain (9), UK (4), Turkey (1), China (18), Australia (6)	XIIa (56)
Goat	1	Algeria (1)	XIIa (1)
Yak	3	China (3)	XIIa (3)
Buffalo	1	South Africa (1)	XIIa (1)
Alpaca	1	Peru (1)	XIIa (1)
Swamp deer	3	Nepal (3)	XIIa (3)
Impala	1	South. Africa (1)	XIIa (1)
Blesbok	1	Czech Republic (1)	XIIa (1)
Nyala	1	Czech Republic (1)	XIIa (1)
Nonhuman primate			
Verreaux’s sifaka	1	USA (1)	XIIb (1)
Human	41	USA (25)	XIIb (9), XIIc (4), XIId (12)
		UK (13)	XIIa (8), XIIb (3), XIId (2)
		Turkey (1)	XIIa (1)
		Peru (1)	XIIa (1)
		Canada (1)	XIIa (1)
Water			
Storm water	15	USA (15)	XIIb (15)
Source water	3	USA (3)	XIIb (2), XIId (1)

*Experimentally infected with specimen from a porcupine.

### Subtyping Marker

To identify a subtyping marker for *C. ubiquitum*, we sequenced the genome of an isolate from a specimen (33496) from a Verreaux’s sifaka by 454 technology using a GS FLX+ System (454 Life Sciences, Branford, CT, USA). This specimen was selected for whole-genome sequencing because of the high number of oocysts present, the availability of ample fecal materials for isolation of oocysts by sucrose and cesium chloride gradient flotation and immunomagnetic separation, and minor contamination from nontarget organisms in extracted DNA. Of the 3,030 assembled contigs of 11.4 MB nucleotides generated from 1,069,468 sequence reads, 1contig (no. 0067), consisting of 45,014 bp, had a high sequence similarity to the 5′ and 3′ ends of the gp60 gene and the flanking intergenic regions. Alignment of the contig 0067 sequence with the nucleotide sequences of the *C. parvum* gp60 gene (AF203016 and AY048665) led to the identification of sequences conserved between *C. ubiquitum* and *C. parvum*, which were used to design a nested PCR that amplified the entire coding region of the gp60 gene, except for the 54 nt at the 3′ end. The sequences of primers used in primary and secondary PCR were 5′-TTTACCCACACATCTGTAGCGTCG-3′ (Ubi-18S-F1) and 5′-ACGGACGGAATGATGTATCTGA-3′ (Ubi-18S-R1), and 5′-ATAGGTGATAATTAGTCAGTCTTTAAT-3′ (Ubi-18S-F2) and 5′-TCCAAAAGCGGCTGAGTCAGCATC-3′ (Ubi-18S-R2), which amplified an expected PCR product of 1,044 and 948 bp, respectively.

### PCR

The partial *C. ubiquitum* gp60 gene was amplified by nested PCR in a total volume of 50 μL, containing 1 μL of DNA (primary PCR) or 2 μL of the primary PCR product (secondary PCR), primers at a concentration of 0.25 µM (Ubi-18S-F1 and Ubi-18S-R1) or 0.5 µM (Ubi-18S-F2 and Ubi-18S-R2), 0.2 µM deoxyribonucleotide triphosphate mix (Promega, Madison, WI, USA), 3 µM MgCl_2_ (Promega), 1 × GeneAmp PCR buffer (Applied Biosystems, Foster City, CA, USA), and 1.25 U of Taq DNA polymerase (Promega). The primary PCR reactions also contained 400 ng/μL nonacetylated bovine serum albumin (Sigma, St. Louis, MO, USA) to reduce PCR inhibition. PCR amplification consisted of an initial denaturation at 94°C for 5 min; 35 cycles at 94°C for 45 s, 45 s at 58°C (primary PCR) or at 55°C (secondary PCR), 1 min at 72°C; and a final extension for 7 min at 72°C. Both positive (DNA from the sifaka specimen) and negative (reagent-grade water) controls were used in each PCR run.

### DNA Sequence Analysis

Products of the secondary gp60 PCR were sequenced in both directions on an ABI 3130 Genetic Analyzer (Applied Biosystems). The sequences were assembled by using ChromasPro 1.32 (www.technelysium.com.au/ChromasPro.html), edited by using BioEdit 7.04 (www.mbio.ncsu.edu/BioEdit/bioedit.html), and aligned by using ClustalX 2.1 (www.clustal.org/). To assess the genetic relatedness of various subtype families of *C. ubiquitum*, we constructed a neighbor-joining tree by using MEGA 5.05 (www.megasoftware.net/). The reliability of cluster formation was evaluated by the bootstrap method with 1,000 replicates. To assess potential recombination among various subtype families, we used DnaSP 5.10 (www.ub.es/dnasp/) to calculate recombination rates on the basis of segregating sites (excluding insertions and deletions). Unique nucleotide sequences derived from this work were deposited in GenBank under accession nos. JX412915–JX412926 and KC204979–KC204985.

### Statistical Analysis

We compared the difference in the distribution of *C. ubiquitum* XIIa and non-XIIa subtype families between rodents and ruminants and between humans in the United States and the United Kingdom by using a χ^2^ test implemented in SPSS 20.0 for Windows software (IBM Corp., Armonk, NY, USA). The difference was considered significant when the p value obtained was <0.05.

## Results

### Features of the gp60 Gene of *C. ubiquitum*

**Figure 1:**
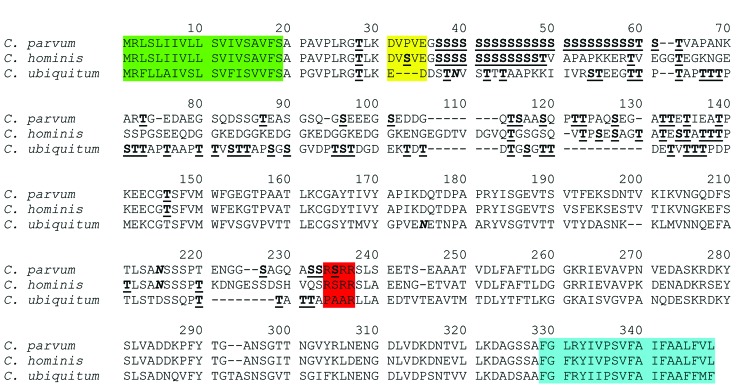
Deduced amino acid sequence of the gp60 gene of *Cryptosporidium ubiquitum* compared with sequences of *C. parvum* and *C. hominis*. gp60 sequences from *C. ubiquitum*, *C. parvum* (GenBank accession no. AF022929), and *C. hominis* (GenBank accession no. ACQ82748) were aligned by using ClustalX (www.clustal.org/). Potential N-linked glycosylation sites are indicated in boldface and italic type, and predicted O-linked glycosylation sites are indicated in boldface and underlined type. The first 19 aa coding for a signal peptide are highlighted in green, and the last 20 aa for a transmembrane domain are highlighted in blue. The signal peptide cleavage site in the N terminal and furin proteolytic cleavage site between gp40 and gp15 of *C. parvum* and *C. hominis* are highlighted in yellow and red, respectively. The furin cleavage site is not present in *C. ubiquitum*. Dashes denote nucleotide deletions.

The gp60 gene in contig 0067 in the whole-genome sequencing of specimen 33496 was 945 bp in length, coding for a peptide that consisted of 315 aa. Except for the 5′ and 3′ regions, the gp60 gene of *C. ubiquitum* had no obvious similarity to the gp60 genes of *C. parvum* and *C. hominis* at the nucleotide level. At the amino acid level, the sequences shared 43%–48% sequence identity with those of *C. parvum* and *C. hominis* (Figure 1). The gene has the same structure of gp60 in *C. parvum* and *C. hominis*, with the first 19 aa coding for a signal peptide and the last 20 aa for a transmembrane domain. As in *C. parvum* and *C. hominis*, upstream of the transmembrane domain, the *C. ubiquitum* gp60 gene sequence had a C-terminus hydrophobic region that is likely linked to a glycosylphosphatidylinositol anchor, 2 potential N-linked glycosylation sites, and numerous O-linked glycosylation sites (*26*,*27*). However, no TCA/TCG/TCT trinucleotide repeats were seen in the 5′ region of the gp60 gene of *C. parvum*, *C. hominis,* and related species (*28*), and no N-terminal amino acids, DVSVE, were found between the putative cleavage site of the signal peptide and trinucleotide repeats (*27*). The putative furin cleavage site RSRR or KSISKR between gp40 and gp15 of *C. parvum* and *C. hominis* (*29*) was not found in *C. ubiquitum* (Figure 1).

### Amplification of gp60 gene of *C. ubiquitum*

Of the 188 DNA preparations of *C. ubiquitum*, 149 yielded PCR products of the expected size, and 145 were successfully sequenced, including 86 from animals, 41 from humans, and 18 from water (Table 1). Nucleotide sequences of isolates from 31 specimens were identical to those of the *C. ubiquitum* reference sequence (contig 0067 from specimen 33496). The remaining sequences had nucleotide differences of 8.2%–38.2% from the reference (Table 2). The gp60 nt sequences of *C. ubiquitum* differed from each other in length at most by 171 bp, with all insertions/deletions in trinucleotide, thus maintaining the reading frame (Technical Appendix Figure). Most of the length differences were due to 4 sequences generated from rodents in the Slovak Republic. The remaining sequences differed from each other in length by at most 12 bp. Most nucleotide sequence polymorphism occurred in the 3′ half of the gene (Technical Appendix).

**Table 2:** Nucleotide sequence similarity (lower triangular matrix) and potential genetic recombination events (upper triangular matrix) among *Cryptosporidium ubiquitum* subtype families at the gp60 locus*

	XIIa	XIIb	XIIc	XIId	XIIe	XIIf
XIIa		1	1	1	1	1
XIIb	87.2		0	0	0	0
XIIc	89.5	91.8		0	0	0
XIId	87.2	90.0	94.5		0	0
XIIe	63.1	69.6	62.2	60.6		0
XIIf	71.5	61.8	70.7	69.5	70.9	

*Nucleotide sequence similarity is indicated by the percentage identity between each pair of sequence compared.

### Nomenclature of *C. ubiquitum* Subtype Families

**Figure 2:**
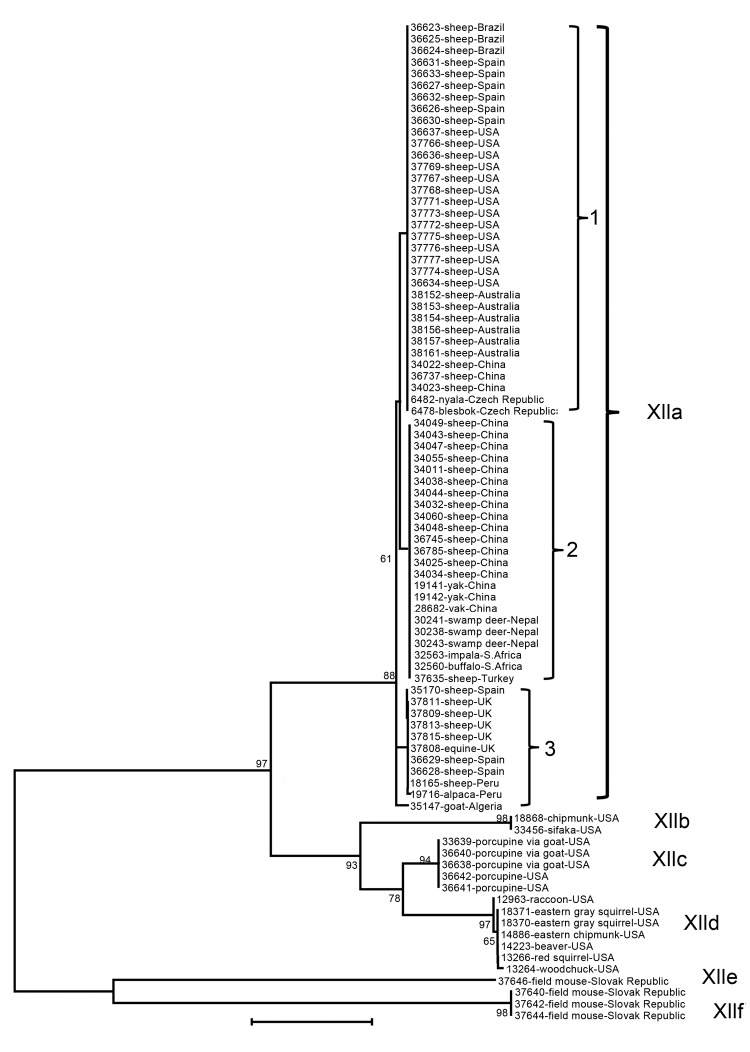
Genetic relationship among 6 *Cryptosporidium ubiquitum* subtype families (XIIa–XIIf) in animals as indicated by a neighbor-joining analysis of the partial gp60 gene. The XIIa subtype family contains all specimens from domestic and wild ruminants, whereas the remaining subtype families contain all specimens from rodents and other wildlife. Within the XIIa subtype family, 1, 2, and 3 denote subtypes 1, 2, and 3, which differ from each other by a few nucleotides. Bootstrap values are indicated along branches. Scale bar indicates 0.02 nucleotide substitutions per site.

The nucleotide sequences of the gp60 gene of *C. ubiquitum* formed 6 subtype families in a neighbor-joining analysis (Figure 2). They were named XIIa, XIIb, XIIc, XIId, XIIe, and XIIf, in concordance with the established nomenclature of gp60 subtype families (*24*). Subtype families XIIe and XIIf formed a cluster highly divergent from the dominant cluster of the 4 subtype families of XIIa, XIIb, XIIc, and XIId (Figure 2). In the dominant cluster, XIIc and XIId diverged at the 3′ end, and the 3′ end of the gene of XIIc had a high identity to XIIa, an indication of possible genetic recombination at the gp60 locus (Technical Appendix). Indeed, DnaSP analysis (www.ub.edu/dnasp/) revealed a minimum of 23 potential recombination events among the 6 subtype families (XIIa–XIIf), and 7 recombination events among the 4 subtype families (XIIa–XIId) in the dominant *C. ubiquitum* cluster. Pairwise recombination event comparisons revealed that the genetic recombination was mostly between XIIa and other subtype families (Table 2).

Within the XIIa subtype, minor sequence differences were found among specimens, leading to the formation of 3 common subtypes (subtypes 1–3) and several rare subtypes with a single nucleotide substitution each (for example, 19716, 35147, 37827, 37828, and 37830). A few nucleotide substitutions were also seen in the subtype family XIId (Technical Appendix). These subtypes within subtype families XIIa and XIId could not be named using the subtype nomenclature based on the number of trinucleotide repeats TCA, TCG, and TGT (*24*) because of the lack of such repeats in the gp60 gene of *C. ubiquitum* (Technical Appendix).

### Subtype Families in Animals

Among 86 isolates from animal specimens characterized, all 68 isolates from Old and New World ruminants belonged to the XIIa subtype family, including 56 from ovine specimens from the United States, Peru, Brazil, Spain, the United Kingdom, Turkey, China, and Australia; 1 from a goat specimen from Algeria; 3 from yak specimens from China; 1 from an alpaca specimen from Peru; and 7 from 5 species of wild ruminants from Nepal, the Czech Republic, and South Africa. One isolate from an equine specimen from the United Kingdom also belonged to the XIIa subtype family. In contrast, XIIb, XIIc, and XIId subtypes were all seen in specimens from several species of rodents and a few other wildlife species (1 raccoon and 1Verreaux’s sifaka) in the United States, and XIIe and XIIf were seen in specimens from field mice in the Slovak Republic (Table 1, Figure 2). The difference in the distribution of XIIa and non-XIIa subtype families between ruminants and rodents was significant (χ^2^ = ∞; p <0.001). This was also the case when the comparison was made with only specimens from the United States (χ^2^ = 26.00; p<0.001).

### Subtype Families in Humans

**Figure 3:**
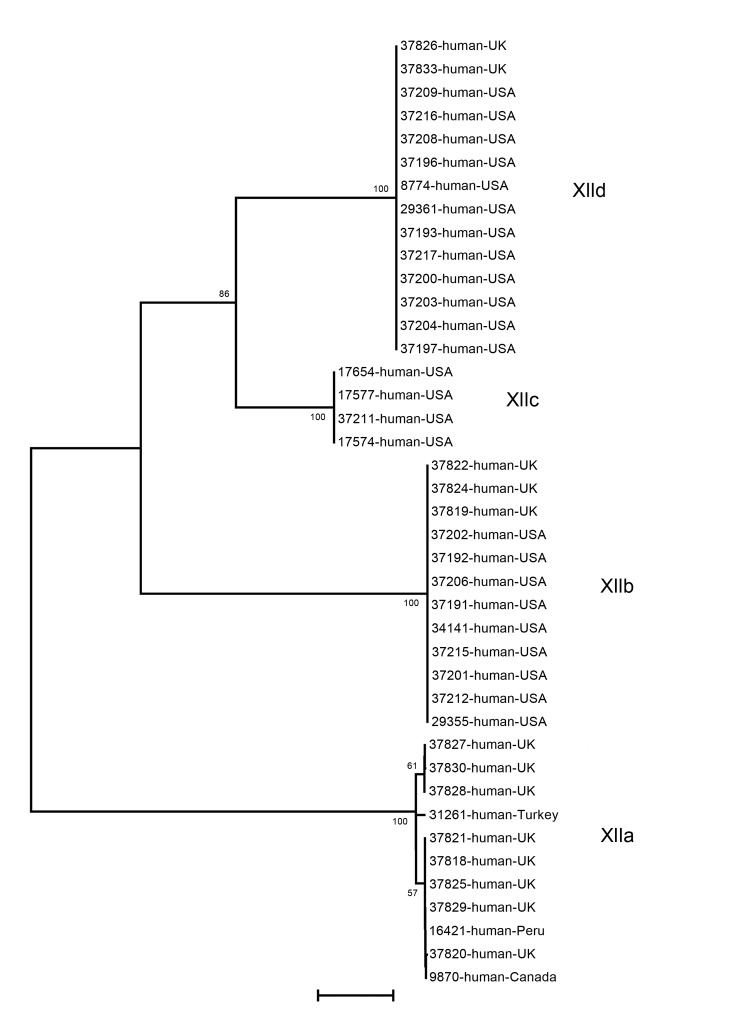
Genetic relationship among 4 *Cryptosporidium ubiquitum* subtype families (XIIa–XIId) in humans as indicated by a neighbor-joining analysis of the partial gp60 gene. The XIIa subtype family is seen in humans in most locations except the United States, where humans are infected with subtype families XIIb, XIIc, and XIId. Bootstrap values are indicated along branches. Scale bar indicates 0.01 nucleotide substitutions per site.

Among 41 isolates from human specimens successfully subtyped, 25 from the United States belonged to the subtype families of XIIb (*9*), XIIc (*4*), and XIId (*12*); 13 isolates from the United Kingdom belonged to the subtype families of XIIa (*8*), XIIb (*3*), and XIId (*2*); and 3 isolates from Turkey, Peru, and Canada belonged to the XIIa subtype family (Table 1; Figure 3). The difference between the distribution of XIIa and non-XIIa subtype families in humans the United States and United Kingdom was significant (χ^2^ = 19.49; p<0.001).

### Subtype Families in Water

Among 18 water samples from the United States, all 15 storm water samples were collected from a drinking source watershed, and 2 river water samples had the XIIb subtype family. Another river water sample revealed organisms from the XIId subtype family (Table 1).

## Discussion

The gp60 gene currently is the most commonly used genetic marker for subtyping *Cryptosporidium* spp., including several notable species that are pathogenic to humans and a few pathogenic species/genotypes that are not found in humans. These gp60-based tools have been used effectively in epidemiologic studies of cryptosporidiosis transmission in humans and farm animals (*2*). However, identifying the gp60 gene of the major emerging human-pathogenic species, *C. ubiquitum*, has been challenging (*24*). In this study, we utilized recent developments in next-generation sequencing technology and conducted a genomic survey of 1 *C. ubiquitum* specimen to identify its gp60 gene. On the basis of results from this sequence survey, we developed a gp60-based *C. ubiquitum* subtyping tool. The application of this new tool in the analysis of specimens from humans, animals, and water has shown the existence of host adaptation in *C. ubiquitum* infections, and the findings suggest that sheep and rodents are a key source of *C. ubiquitum* transmission to humans, possibly through direct human contact with infected animals or by contamination of drinking source water.

The gp60 gene of *C. ubiquitum* has extensive sequence differences from the gp60 gene of other *Cryptosporidium* spp. Nucleotide sequences of the near complete gp60 gene obtained from the 6 *C. ubiquitum* subtype families all showed extremely low identity with those of *C. parvum*, *C. hominis*, and related species. Even in the primer regions, substantial nucleotide differences occurred between *C. ubiquitum* and other species/genotypes. These nucleotide sequence differences could be responsible for the inability of commonly used gp60 primers that were designed on the basis of *C. parvum* and *C. hominis* sequences, to amplify the *C. ubiquitum* gp60 gene (*24*).

The sequence differences between the gp60 gene in *C. ubiquitum* and the gp60 gene in other *Cryptosporidium* spp. may affect its functions. Unlike the gp60 gene of all 11 previously characterized *Cryptosporidium* spp. and genotypes (*24*), the trinucleotide repeats of TCA/TCG/TCT, which code for the polyserine tract at the 5′end of the gene, commonly used to differentiate subtypes within each subtype family, were not observed in the gp60 gene sequence of *C. ubiquitum*. Previous research suggested that subtypes with short serine repeats may be selectively favored in humans over long repeats (*23*). Other unique features of the gp60 gene in *C. ubiquitum* include the absence of the signal peptide cleavage site in the N terminal and furin proteolytic cleavage site between gp40 and gp15. These differences could affect the processing and transport of the gp60 protein.

Although *C. ubiquitum* has the most broad host range among *Cryptosporidium* spp., the subtype data generated provide evidence that infection with this species has led to host adaptation. All field specimens from domestic and wild ruminants in both the New and Old World belonged to the XIIa subtype family. Despite its common occurrence in both domestic and wild ruminants in many geographic areas, subtype family XIIa has not been found in rodents in the United States and the Slovak Republic. Nevertheless, this host adaptation is not strict host specificity; *C. ubiquitum* of the XIIc subtype family was experimentally transmitted from a ring-tailed porcupine to goats, and the XIIa subtype family was found in an isolate from 1 equine specimen from the United Kingdom (Table 1 and [*12*]).

In contrast to the US findings, subtype family XIIa appears to be more commonly seen in *C. ubiquitum* isolates that infect humans in other areas. Thus, 8 of 13 cases of human infection in the United Kingdom and all 3 cases in humans from 3 other countries were caused by isolates of the subtype family XIIa. The source of *C. ubiquitum* infections in humans is not entirely clear. Because of the common occurrence of the XIIa subtype family in sheep, contact with sheep could be a frequent source of human infection in the United Kingdom and other industrialized nations. Indeed, among the 8 persons infected with XIIa in the United Kingdom, 4 had contact with sheep, 2 with so-called farm animals, and 1 with dogs. The remaining 5 case-patients with infections caused by subtypes XIIb and XIId in the United Kingdom reported no contact with sheep, although 4 reported contact with pets (dogs, cats, or pet birds), which are not known hosts of *C. ubiquitum*. One case-patient who was infected with *C. ubiquitum* of the XIIb subtype reported swimming in pools and had sick family members, indicating potential acquisition of the infection through another transmission route.

Drinking untreated water contaminated by wildlife might be a potential source of *C. ubiquitum* infections in the United States. All US *C. ubiquitum* specimens from humans characterized in this study belonged to the same subtype families found in wild rodents in this country. Because persons in the United States usually have little direct contact with wild rodents, direct zoonotic transmission of *C. ubiquitum* infection is probably less important in this country. This is supported by the absence of the XIIa subtype family in infected humans in the United States. Although XIIa is clearly common in sheep in the United States, the density of sheep is lower than it is in the United Kingdom. *C. ubiquitum* is one of the most common *Cryptosporidium* spp. in drinking source water in the United States (*17*–*19*). In this study, XIIb and XIId were detected in drinking source water in the United States, and most water samples were collected from the same watershed where US rodent specimens had been collected.

In conclusion, data generated thus far have shown host adaptation in *C. ubiquitum* at the gp60 locus and some potential geographic differences in the epidemiology of *C. ubiquitum* infections in humans. These finding highlight the need for subtype analysis of unusual *Cryptosporidium* species to clarify the sources and transmission dynamics of zoonotic cryptosporidiosis in rural areas.

Technical Appendix Nucleotide alignment of the 60-kDa glycoprotein (gp60) gene of *Cryptosporidium ubiquitum*.
