# Supplementary material for: Subtyping Cryptosporidium ubiquitum,a Zoonotic Pathogen Emerging in Humans
Source: Emerg Infect Dis. 2014 Feb;20(2):217–24. doi: 10.3201/eid2002.121797 (PMC3901490; doi:10.3201/eid2002.121797)
Supplement: Technical Appendix — Nucleotide alignment of the 60-kDa glycoprotein (gp60) gene of Cryptosporidium ubiquitum. [file 12-1797-Techapp-s1.pdf]

Article DOI: http://dx.doi.org/10.3201/eid2002.121797

Subtyping *Cryptosporidium ubiquitum,*a Zoonotic Pathogen Emerging in Humans

Technical Appendix

	Sequence (positions 1–100)
	10 20 30 40 50 60 70 80 90 100
	....|....|....|....|....|....|....|....|....|....|....|....|....|....|....|....|....|....|....|....|
36625-sheep-Brazil	TGAGATTTTTACTCGCTATCGTATCACTCTCCGTTTTCATCTCAGTTGTATTCTCAGCTCCAGGTGTTCCACTCAGAGGTACATTGAAAGAGGATGACAG
36624-sheep-Brazil	....
36623-sheep-Brazil	....
36630-sheep-Spain	....
36631-sheep-Spain	....
36633-sheep-Spain	....
36627-sheep-Spain	....
36632-sheep-Spain	....
36626-sheep-Spain	....
36634-sheep-USA	....
36637-sheep-USA	....
37766-sheep-USA	....
36636-sheep-USA	....
37769-sheep-USA	....
37767-sheep-USA	....
37768-sheep-USA	....
37774-sheep-USA	....
37777-sheep-USA	....
37776-sheep-USA	....
37775-sheep-USA	....
37772-sheep-USA	....
37773-sheep-USA	....
37771-sheep-USA	....
6478-blesbok-Czech Republic	....
6482-nyala-Czech Republic	....
34023-sheep-China	....
36737-sheep-China	....
34022-sheep-China	....
38161-sheep-Australia	....
38157-sheep-Australia	....
38156-sheep-Australia	....
38154-sheep-Australia	....
38153-sheep-Australia	....
38152-sheep-Australia	....
34049-sheep-China	....
37635-sheep-Turkey	....
34043-sheep-China	....
34047-sheep-China	....
34055-sheep-China	....
34011-sheep-China	....
34038-sheep-China	....
34044-sheep-China	....
34032-sheep-China	....
34060-sheep-China	....
34048-sheep-China	....
36745-sheep-China	....
36785-sheep-China	....
34025-sheep-China	....
34034-sheep-China	....
19141-yak-China	....
19142-yak-China	....
28682-yak-China	....
30241-swamp deer-Nepal	....
30238-swamp deer-Nepal	....
30243-swamp deer-Nepal	....
32563-impala-S.Africa	....
32560-buffallo-S.Africa	....
31261-human-Turkey	....
37828-human-UK	....
37830-human-UK	....
37827-human-UK	....
31570-sheep-Spain	....
37820-human-UK	....
37811-sheep-UK	....
37809-sheep-UK	....
37813-sheep-UK	....
37815-sheep-UK	....
37808-equine-UK	....
36629-sheep-Spain	....
36628-sheep-Spain	....
18165-sheep-Peru	....
37821-human-UK	....
37818-human-UK	....
37825-human-UK	....
37829-human-UK	....
16421-human-Peru	....
9870-human-Canada	....
19716-alpaca-Peru	....
35147-goat-Algeria	....
18868-chipmunk-USA	....
33456-sifaka-USA	....
37822-human-UK	....
37824-human-UK	....
37819-human-UK	....
37202-human-USA	....
37192-human-USA	....
37206-human-USA	....
37191-human-USA	....
34141-human-USA	....
37215-human-USA	....
37201-human-USA	....
37212-human-USA	....
29355-human-USA	....
8512-storm-water-USA	....
8513-storm-water-USA	....
5397-storm-water-USA	....
6140-storm-water-USA	....
8509-storm-water-USA	....
8469-storm-water-USA	....
6141-storm-water-USA	....
8062-storm-water-USA	....

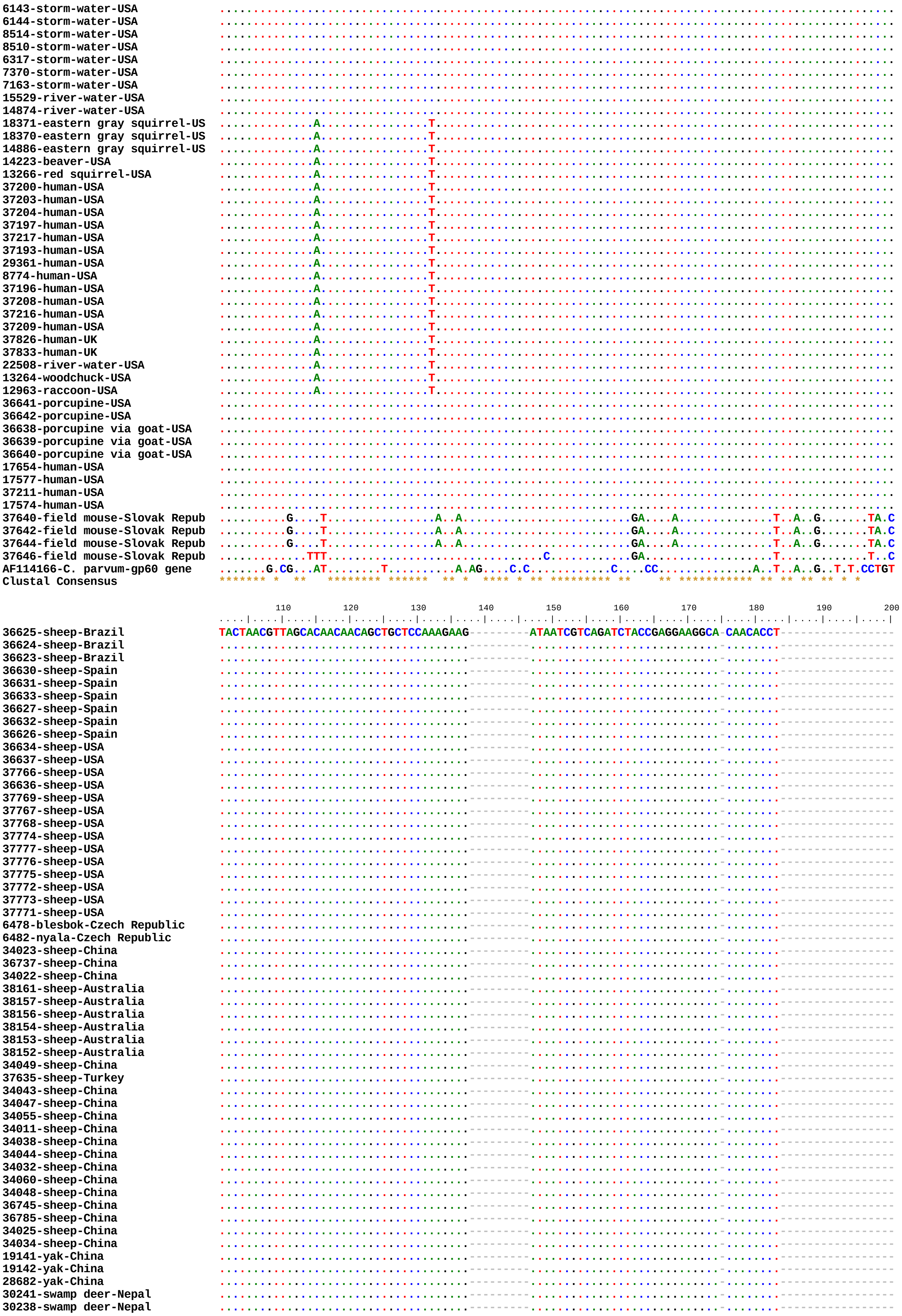
6143-storm-water-USA
6144-storm-water-USA
8514-storm-water-USA
8510-storm-water-USA
6317-storm-water-USA
7370-storm-water-USA
7163-storm-water-USA
15529-river-water-USA
14874-river-water-USA
18371-eastern gray squirrel-US
18370-eastern gray squirrel-US
14886-eastern gray squirrel-US
14223-beaver-USA
13266-red squirrel-USA
37200-human-USA
37203-human-USA
37204-human-USA
37197-human-USA
37217-human-USA
37193-human-USA
29361-human-USA
8774-human-USA
37196-human-USA
37208-human-USA
37216-human-USA
37209-human-USA
37826-human-UK
37833-human-UK
22508-river-water-USA
13264-woodchuck-USA
12963-raccoon-USA
36641-porcupine-USA
36642-porcupine-USA
36638-porcupine via goat-USA
36639-porcupine via goat-USA
36640-porcupine via goat-USA
17654-human-USA
17577-human-USA
37211-human-USA
17574-human-USA
37640-field mouse-Slovak Repub
37642-field mouse-Slovak Repub
37644-field mouse-Slovak Repub
37646-field mouse-Slovak Repub
AF114166-C. parvum-gp60 gene
Clustal Consensus
110 120 130 140 150 160 170 180 190 200
36625-sheep-Brazil TACTAACGTTAGCACAACAACAGCTGCTCCAAAGAAG-------ATAATCGTCAGATCTACCGAGGAAGGCA-CAACACCT
36624-sheep-Brazil
36623-sheep-Brazil
36630-sheep-Spain
36631-sheep-Spain
36633-sheep-Spain
36627-sheep-Spain
36632-sheep-Spain
36626-sheep-Spain
36634-sheep-USA
36637-sheep-USA
37766-sheep-USA
36636-sheep-USA
37769-sheep-USA
37767-sheep-USA
37768-sheep-USA
37774-sheep-USA
37777-sheep-USA
37776-sheep-USA
37775-sheep-USA
37772-sheep-USA
37773-sheep-USA
37771-sheep-USA
6478-blesbok-Czech Republic
6482-nyala-Czech Republic
34023-sheep-China
36737-sheep-China
34022-sheep-China
38161-sheep-Australia
38157-sheep-Australia
38156-sheep-Australia
38154-sheep-Australia
38153-sheep-Australia
38152-sheep-Australia
34049-sheep-China
37635-sheep-Turkey
34043-sheep-China
34047-sheep-China
34055-sheep-China
34011-sheep-China
34038-sheep-China
34044-sheep-China
34032-sheep-China
34060-sheep-China
34048-sheep-China
36745-sheep-China
36785-sheep-China
34025-sheep-China
34034-sheep-China
19141-yak-China
19142-yak-China
28682-yak-China
30241-swamp deer-Nepal
30238-swamp deer-Nepal

```
30243-swamp deer-Nepal          .....................................---------............................-........-----------------
32563-impala-S.Africa           .....................................---------............................-........-----------------
32560-buffalo-S.Africa          .....................................---------............................-........-----------------
31261-human-Turkey              .....................................---------............................-........-----------------
37828-human-UK                  .....................................---------............................-........-----------------
37830-human-UK                  .....................................---------............................-........-----------------
37827-human-UK                  .....................................---------............................-........-----------------
31570-sheep-Spain               .....................................---------............................-........-----------------
37820-human-UK                  .....................................---------............................-........-----------------
37811-sheep-UK                  .....................................---------............................-........-----------------
37809-sheep-UK                  .....................................---------............................-........-----------------
37813-sheep-UK                  .....................................---------............................-........-----------------
37815-sheep-UK                  .....................................---------............................-........-----------------
37808-equine-UK                 .....................................---------............................-........-----------------
36629-sheep-Spain               .....................................---------............................-........-----------------
36628-sheep-Spain               .....................................---------............................-........-----------------
18165-sheep-Peru                .....................................---------............................-........-----------------
37821-human-UK                  .....................................---------............................-........-----------------
37818-human-UK                  .....................................---------............................-........-----------------
37825-human-UK                  .....................................---------............................-........-----------------
37829-human-UK                  .....................................---------............................-........-----------------
16421-human-Peru                .....................................---------............................-........-----------------
9870-human-Canada               .....................................---------............................-........-----------------
19716-alpaca-Peru               .....................................---------............................-........-----------------
35147-goat-Algeria              .....................................---------............................-........-----------------
18868-chipmunk-USA              .....................................---------............................-........-----------------
33456-sifaka-USA                .....................................---------............................-........-----------------
37822-human-UK                  .....................................---------............................-........-----------------
37824-human-UK                  .....................................---------............................-........-----------------
37819-human-UK                  .....................................---------............................-........-----------------
37202-human-USA                 .....................................---------............................-........-----------------
37192-human-USA                 .....................................---------............................-........-----------------
37206-human-USA                 .....................................---------............................-........-----------------
37191-human-USA                 .....................................---------............................-........-----------------
34141-human-USA                 .....................................---------............................-........-----------------
37215-human-USA                 .....................................---------............................-........-----------------
37201-human-USA                 .....................................---------............................-........-----------------
37212-human-USA                 .....................................---------............................-........-----------------
29355-human-USA                 .....................................---------............................-........-----------------
8512-storm-water-USA            .....................................---------............................-........-----------------
8513-storm-water-USA            .....................................---------............................-........-----------------
5397-storm-water-USA            .....................................---------............................-........-----------------
6140-storm-water-USA            .....................................---------............................-........-----------------
8509-storm-water-USA            .....................................---------............................-........-----------------
8469-storm-water-USA            .....................................---------............................-........-----------------
6141-storm-water-USA            .....................................---------............................-........-----------------
8062-storm-water-USA            .....................................---------............................-........-----------------
6143-storm-water-USA            .....................................---------............................-........-----------------
6144-storm-water-USA            .....................................---------............................-........-----------------
8514-storm-water-USA            .....................................---------............................-........-----------------
8510-storm-water-USA            .....................................---------............................-........-----------------
6317-storm-water-USA            .....................................---------............................-........-----------------
7370-storm-water-USA            .....................................---------............................-........-----------------
7163-storm-water-USA            .....................................---------............................-........-----------------
15529-river-water-USA           .....................................---------............................-........-----------------
14874-river-water-USA           .....................................---------............................-........-----------------
18371-eastern gray squirrel-US  .....................................---------............................-........-----------------
18370-eastern gray squirrel-US  .....................................---------............................-........-----------------
14886-eastern gray squirrel-US  .....................................---------............................-........-----------------
14223-beaver-USA                .....................................---------............................-........-----------------
13266-red squirrel-USA          .....................................---------............................-........-----------------
37200-human-USA                 .....................................---------............................-........-----------------
37203-human-USA                 .....................................---------............................-........-----------------
37204-human-USA                 .....................................---------............................-........-----------------
37197-human-USA                 .....................................---------............................-........-----------------
37217-human-USA                 .....................................---------............................-........-----------------
37193-human-USA                 .....................................---------............................-........-----------------
29361-human-USA                 .....................................---------............................-........-----------------
8774-human-USA                  .....................................---------............................-........-----------------
37196-human-USA                 .....................................---------............................-........-----------------
37208-human-USA                 .....................................---------............................-........-----------------
37216-human-USA                 .....................................---------............................-........-----------------
37209-human-USA                 .....................................---------............................-........-----------------
37826-human-UK                  .....................................---------............................-........-----------------
37833-human-UK                  .....................................---------............................-........-----------------
22508-river-water-USA           .....................................---------............................-........-----------------
13264-woodchuck-USA             ...............G.....................---------............................-........-----------------
12963-raccoon-USA               .....................................---------............................-........-----------------
36641-porcupine-USA             .....................................---------............................-........-----------------
36642-porcupine-USA             .....................................---------............................-........-----------------
36638-porcupine via goat-USA    .....................................---------............................-........-----------------
36639-porcupine via goat-USA    .....................................---------............................-........-----------------
36640-porcupine via goat-USA    .....................................---------............................-........-----------------
17654-human-USA                 .....................................---------............................-........-----------------
17577-human-USA                 .....................................---------............................-........-----------------
37211-human-USA                 .....................................---------............................-........-----------------
17574-human-USA                 .....................................---------............................-........-----------------
37640-field mouse-Slovak Repub  C.GCG.TAGCTCG.....C..................---------.................T..A.....T.-..G..GT.GCTGGTGCTACTCCAAC
37642-field mouse-Slovak Repub  C.GCG.TAGCTCG.....C..................---------.................T..A.....T.-..G..GT.GCTGGTGCTACTCC---
37644-field mouse-Slovak Repub  C.GCG.TAGCTCG.....C..................---------.................T..A.....T.-..G..GT.GCTGGTGCTACTCCAAC
37646-field mouse-Slovak Repub  .GG.G.TAGC.CG.....T..................---------.................A..A.....T.-..GTTGT.CCAGGTGCTGCT-----
AF114166-C. parvum-gp60 gene    .GAGGG.-.C.T..TCGTC.TC.TCATC------------------..C...A...TCATC.T.ATCATCAT..T.....T.------------------
Clustal Consensus                            *     *  *                        ** *** ***     *           * **

                                         210        220        230        240        250        260        270        280        290        300
                                ....|....|....|....|....|....|....|....|....|....|....|....|....|....|....|....|....|....|....|....|
36625-sheep-Brazil              -------------------------------G--CTCCT-------------ACTACTCCTAG--------------------TACTACT----------
36624-sheep-Brazil              -------------------------------.--.....-------------...........--------------------.......----------
36623-sheep-Brazil              -------------------------------.--.....-------------...........--------------------.......----------
36630-sheep-Spain               -------------------------------.--.....-------------...........--------------------.......----------
36631-sheep-Spain               -------------------------------.--.....-------------...........--------------------.......----------
36633-sheep-Spain               -------------------------------.--.....-------------...........--------------------.......----------
36627-sheep-Spain               -------------------------------.--.....-------------...........--------------------.......----------
```

```
36632-sheep-Spain               -------------------------------.---.....------------...........--------------------.......----------
36626-sheep-Spain               -------------------------------.---.....------------...........--------------------.......----------
36634-sheep-USA                 -------------------------------.---.....------------...........--------------------.......----------
36637-sheep-USA                 -------------------------------.---.....------------...........--------------------.......----------
37766-sheep-USA                 -------------------------------.---.....------------...........--------------------.......----------
36636-sheep-USA                 -------------------------------.---.....------------...........--------------------.......----------
37769-sheep-USA                 -------------------------------.---.....------------...........--------------------.......----------
37767-sheep-USA                 -------------------------------.---.....------------...........--------------------.......----------
37768-sheep-USA                 -------------------------------.---.....------------...........--------------------.......----------
37774-sheep-USA                 -------------------------------.---.....------------...........--------------------.......----------
37777-sheep-USA                 -------------------------------.---.....------------...........--------------------.......----------
37776-sheep-USA                 -------------------------------.---.....------------...........--------------------.......----------
37775-sheep-USA                 -------------------------------.---.....------------...........--------------------.......----------
37772-sheep-USA                 -------------------------------.---.....------------...........--------------------.......----------
37773-sheep-USA                 -------------------------------.---.....------------...........--------------------.......----------
37771-sheep-USA                 -------------------------------.---.....------------...........--------------------.......----------
6478-blesbok-Czech Republic     -------------------------------.---.....------------...........--------------------.......----------
6482-nyala-Czech Republic       -------------------------------.---.....------------...........--------------------.......----------
34023-sheep-China               -------------------------------.---.....------------...........--------------------.......----------
36737-sheep-China               -------------------------------.---.....------------...........--------------------.......----------
34022-sheep-China               -------------------------------.---.....------------...........--------------------.......----------
38161-sheep-Australia           -------------------------------.---.....------------...........--------------------.......----------
38157-sheep-Australia           -------------------------------.---.....------------...........--------------------.......----------
38156-sheep-Australia           -------------------------------.---.....------------...........--------------------.......----------
38154-sheep-Australia           -------------------------------.---.....------------...........--------------------.......----------
38153-sheep-Australia           -------------------------------.---.....------------...........--------------------.......----------
38152-sheep-Australia           -------------------------------.---.....------------...........--------------------.......----------
34049-sheep-China               -------------------------------.---.....------------...........--------------------.......----------
37635-sheep-Turkey              -------------------------------.---.....------------...........--------------------.......----------
34043-sheep-China               -------------------------------.---.....------------...........--------------------.......----------
34047-sheep-China               -------------------------------.---.....------------...........--------------------.......----------
34055-sheep-China               -------------------------------.---.....------------...........--------------------.......----------
34011-sheep-China               -------------------------------.---.....------------...........--------------------.......----------
34038-sheep-China               -------------------------------.---.....------------...........--------------------.......----------
34044-sheep-China               -------------------------------.---.....------------...........--------------------.......----------
34032-sheep-China               -------------------------------.---.....------------...........--------------------.......----------
34060-sheep-China               -------------------------------.---.....------------...........--------------------.......----------
34048-sheep-China               -------------------------------.---.....------------...........--------------------.......----------
36745-sheep-China               -------------------------------.---.....------------...........--------------------.......----------
36785-sheep-China               -------------------------------.---.....------------...........--------------------.......----------
34025-sheep-China               -------------------------------.---.....------------...........--------------------.......----------
34034-sheep-China               -------------------------------.---.....------------...........--------------------.......----------
19141-yak-China                 -------------------------------.---.....------------...........--------------------.......----------
19142-yak-China                 -------------------------------.---.....------------...........--------------------.......----------
28682-yak-China                 -------------------------------.---.....------------...........--------------------.......----------
30241-swamp deer-Nepal          -------------------------------.---.....------------...........--------------------.......----------
30238-swamp deer-Nepal          -------------------------------.---.....------------...........--------------------.......----------
30243-swamp deer-Nepal          -------------------------------.---.....------------...........--------------------.......----------
32563-impala-S.Africa           -------------------------------.---.....------------...........--------------------.......----------
32560-buffalo-S.Africa          -------------------------------.---.....------------...........--------------------.......----------
31261-human-Turkey              -------------------------------.---.....------------...........--------------------.......----------
37828-human-UK                  -------------------------------.---.....------------...........--------------------.......----------
37830-human-UK                  -------------------------------.---.....------------...........--------------------.......----------
37827-human-UK                  -------------------------------.---.....------------...........--------------------.......----------
31570-sheep-Spain               -------------------------------.---.....------------...........--------------------.......----------
37820-human-UK                  -------------------------------.---.....------------...........--------------------.......----------
37811-sheep-UK                  -------------------------------.---.....------------...........--------------------.......----------
37809-sheep-UK                  -------------------------------.---.....------------...........--------------------.......----------
37813-sheep-UK                  -------------------------------.---.....------------...........--------------------.......----------
37815-sheep-UK                  -------------------------------.---.....------------...........--------------------.......----------
37808-equine-UK                 -------------------------------.---.....------------...........--------------------.......----------
36629-sheep-Spain               -------------------------------.---.....------------...........--------------------.......----------
36628-sheep-Spain               -------------------------------.---.....------------...........--------------------.......----------
18165-sheep-Peru                -------------------------------.---.....------------...........--------------------.......----------
37821-human-UK                  -------------------------------.---.....------------...........--------------------.......----------
37818-human-UK                  -------------------------------.---.....------------...........--------------------.......----------
37825-human-UK                  -------------------------------.---.....------------...........--------------------.......----------
37829-human-UK                  -------------------------------.---.....------------...........--------------------.......----------
16421-human-Peru                -------------------------------.---.....------------...........--------------------.......----------
9870-human-Canada               -------------------------------.---.....------------...........--------------------.......----------
19716-alpaca-Peru               -------------------------------.---.....------------...........--------------------.......----------
35147-goat-Algeria              -------------------------------.---.....------------...........--------------------.......----------
18868-chipmunk-USA              ----------------------------------A..G..CC------TACT...........--------------------.......----------
33456-sifaka-USA                ----------------------------------A..G..CC------TACT...........--------------------.......----------
37822-human-UK                  ----------------------------------A..G..CC------TACT...........--------------------.......----------
37824-human-UK                  ----------------------------------A..G..CC------TACT...........--------------------.......----------
37819-human-UK                  ----------------------------------A..G..CC------TACT...........--------------------.......----------
37202-human-USA                 ----------------------------------A..G..CC------TACT...........--------------------.......----------
37192-human-USA                 ----------------------------------A..G..CC------TACT...........--------------------.......----------
37206-human-USA                 ----------------------------------A..G..CC------TACT...........--------------------.......----------
37191-human-USA                 ----------------------------------A..G..CC------TACT...........--------------------.......----------
34141-human-USA                 ----------------------------------A..G..CC------TACT...........--------------------.......----------
37215-human-USA                 ----------------------------------A..G..CC------TACT...........--------------------.......----------
37201-human-USA                 ----------------------------------A..G..CC------TACT...........--------------------.......----------
37212-human-USA                 ----------------------------------A..G..CC------TACT...........--------------------.......----------
29355-human-USA                 ----------------------------------A..G..CC------TACT...........--------------------.......----------
8512-storm-water-USA            ----------------------------------A..G..CC------TACT...........--------------------.......----------
8513-storm-water-USA            ----------------------------------A..G..CC------TACT...........--------------------.......----------
5397-storm-water-USA            ----------------------------------A..G..CC------TACT...........--------------------.......----------
6140-storm-water-USA            ----------------------------------A..G..CC------TACT...........--------------------.......----------
8509-storm-water-USA            ----------------------------------A..G..CC------TACT...........--------------------.......----------
8469-storm-water-USA            ----------------------------------A..G..CC------TACT...........--------------------.......----------
6141-storm-water-USA            ----------------------------------A..G..CC------TACT...........--------------------.......----------
8062-storm-water-USA            ----------------------------------A..G..CC------TACT...........--------------------.......----------
6143-storm-water-USA            ----------------------------------A..G..CC------TACT...........--------------------.......----------
6144-storm-water-USA            ----------------------------------A..G..CC------TACT...........--------------------.......----------
8514-storm-water-USA            ----------------------------------A..G..CC------TACT...........--------------------.......----------
8510-storm-water-USA            ----------------------------------A..G..CC------TACT...........--------------------.......----------
6317-storm-water-USA            ----------------------------------A..G..CC------TACT...........--------------------.......----------
7370-storm-water-USA            ----------------------------------A..G..CC------TACT...........--------------------.......----------
7163-storm-water-USA            ----------------------------------A..G..CC------TACT...........--------------------.......----------
15529-river-water-USA           ----------------------------------A..G..CC------TACT...........--------------------.......----------
14874-river-water-USA           ----------------------------------A..G..CC------TACT...........--------------------.......----------
```

```
18371-eastern gray squirrel-US ----------------------------------A..------------------........--------------------.......----------
18370-eastern gray squirrel-US ----------------------------------A..------------------........--------------------.......----------
14886-eastern gray squirrel-US ----------------------------------A..------------------........--------------------.......----------
14223-beaver-USA               ----------------------------------A..------------------........--------------------.......----------
13266-red squirrel-USA         ----------------------------------A..------------------........--------------------.......----------
37200-human-USA                ----------------------------------A..------------------........--------------------.......----------
37203-human-USA                ----------------------------------A..------------------........--------------------.......----------
37204-human-USA                ----------------------------------A..------------------........--------------------.......----------
37197-human-USA                ----------------------------------A..------------------........--------------------.......----------
37217-human-USA                ----------------------------------A..------------------........--------------------.......----------
37193-human-USA                ----------------------------------A..------------------........--------------------.......----------
29361-human-USA                ----------------------------------A..------------------........--------------------.......----------
8774-human-USA                 ----------------------------------A..------------------........--------------------.......----------
37196-human-USA                ----------------------------------A..------------------........--------------------.......----------
37208-human-USA                ----------------------------------A..------------------........--------------------.......----------
37216-human-USA                ----------------------------------A..------------------........--------------------.......----------
37209-human-USA                ----------------------------------A..------------------........--------------------.......----------
37826-human-UK                 ----------------------------------A..------------------........--------------------.......----------
37833-human-UK                 ----------------------------------A..------------------........--------------------.......----------
22508-river-water-USA          ----------------------------------A..------------------........--------------------.......----------
13264-woodchuck-USA            ----------------------------------A..------------------........--------------------.......----------
12963-raccoon-USA              ----------------------------------A..------------------........--------------------.......----------
36641-porcupine-USA            ----------------------------------A..G..CC---------T...........--------------------.......----------
36642-porcupine-USA            ----------------------------------A..G..CC---------T...........--------------------.......----------
36638-porcupine via goat-USA   ----------------------------------A..G..CC---------T...........--------------------.......----------
36639-porcupine via goat-USA   ----------------------------------A..G..CC---------T...........--------------------.......----------
36640-porcupine via goat-USA   ----------------------------------A..G..CC---------T...........--------------------.......----------
17654-human-USA                ----------------------------------A..G..CC---------T...........--------------------.......----------
17577-human-USA                ----------------------------------A..G..CC---------T...........--------------------.......----------
37211-human-USA                ----------------------------------A..G..CC---------T...........--------------------.......----------
17574-human-USA                ----------------------------------A..G..CC---------T...........--------------------.......----------
37640-field mouse-Slovak Repub TACTCCTGGTGCAGCAGCAACAACTCCTGGT.CTA.....GGTGCAGCAGCA..A......G.--------------------.G.....CCTGGTGCAG
37642-field mouse-Slovak Repub ------TGGTGCAGCAGCAACAACTCCTGGT.CTA.....GGTGCAGCAGCA..A......G.--------------------.G.....CCTGGTGCAG
37644-field mouse-Slovak Repub TACTCCTGGTGCAGCAGCAACAACTCCTGGT.CTA.....GGTGCAGCAGCA..A......G.--------------------.G.....CCTGGTGCAG
37646-field mouse-Slovak Repub -------------------------------.CTA....C------------..C..C....C--------------------.G.G...----------
AF114166-C. parvum-gp60 gene   ---------------------------------------------AACCGTCG.AC.AG.A.A--------------------..AGG.AAGAACT----
Clustal Consensus                                                                      *  *                       *    *

                                        310       320       330       340       350       360       370       380       390       400
                               ....|....|....|....|....|....|....|....|....|....|....|....|....|....|....|....|....|....|....|....|
36625-sheep-Brazil             --------------G---CTCCTACT---GCTGCTCCCACTACTGTTAGTACTACC------GCTCCTTCTGGCGGCGGTGTTACTC-------------
36624-sheep-Brazil             --------------.---........---...........................------.........................-------------
36623-sheep-Brazil             --------------.---........---...........................------.........................-------------
36630-sheep-Spain              --------------.---........---...........................------.........................-------------
36631-sheep-Spain              --------------.---........---...........................------.........................-------------
36633-sheep-Spain              --------------.---........---...........................------.........................-------------
36627-sheep-Spain              --------------.---........---...........................------.........................-------------
36632-sheep-Spain              --------------.---........---...........................------.........................-------------
36626-sheep-Spain              --------------.---........---...........................------.........................-------------
36634-sheep-USA                --------------.---........---...........................------.........................-------------
36637-sheep-USA                --------------.---........---...........................------.........................-------------
37766-sheep-USA                --------------.---........---...........................------.........................-------------
36636-sheep-USA                --------------.---........---...........................------.........................-------------
37769-sheep-USA                --------------.---........---...........................------.........................-------------
37767-sheep-USA                --------------.---........---...........................------.........................-------------
37768-sheep-USA                --------------.---........---...........................------.........................-------------
37774-sheep-USA                --------------.---........---...........................------.........................-------------
37777-sheep-USA                --------------.---........---...........................------.........................-------------
37776-sheep-USA                --------------.---........---...........................------.........................-------------
37775-sheep-USA                --------------.---........---...........................------.........................-------------
37772-sheep-USA                --------------.---........---...........................------.........................-------------
37773-sheep-USA                --------------.---........---...........................------.........................-------------
37771-sheep-USA                --------------.---........---...........................------.........................-------------
6478-blesbok-Czech Republic    --------------.---........---...........................------.........................-------------
6482-nyala-Czech Republic      --------------.---........---...........................------.........................-------------
34023-sheep-China              --------------.---........---...........................------.........................-------------
36737-sheep-China              --------------.---........---...........................------.........................-------------
34022-sheep-China              --------------.---........---...........................------.........................-------------
38161-sheep-Australia          --------------.---........---...........................------.........................-------------
38157-sheep-Australia          --------------.---........---...........................------.........................-------------
38156-sheep-Australia          --------------.---........---...........................------.........................-------------
38154-sheep-Australia          --------------.---........---...........................------.........................-------------
38153-sheep-Australia          --------------.---........---...........................------.........................-------------
38152-sheep-Australia          --------------.---........---...........................------.........................-------------
34049-sheep-China              --------------.---..T.....---...........................------.........................-------------
37635-sheep-Turkey             --------------.---..T.....---...........................------.........................-------------
34043-sheep-China              --------------.---..T.....---...........................------.........................-------------
34047-sheep-China              --------------.---..T.....---...........................------.........................-------------
34055-sheep-China              --------------.---..T.....---...........................------.........................-------------
34011-sheep-China              --------------.---..T.....---...........................------.........................-------------
34038-sheep-China              --------------.---..T.....---...........................------.........................-------------
34044-sheep-China              --------------.---..T.....---...........................------.........................-------------
34032-sheep-China              --------------.---..T.....---...........................------.........................-------------
34060-sheep-China              --------------.---..T.....---...........................------.........................-------------
34048-sheep-China              --------------.---..T.....---...........................------.........................-------------
36745-sheep-China              --------------.---..T.....---...........................------.........................-------------
36785-sheep-China              --------------.---..T.....---...........................------.........................-------------
34025-sheep-China              --------------.---..T.....---...........................------.........................-------------
34034-sheep-China              --------------.---..T.....---...........................------.........................-------------
19141-yak-China                --------------.---..T.....---...........................------.........................-------------
19142-yak-China                --------------.---..T.....---...........................------.........................-------------
28682-yak-China                --------------.---..T.....---...........................------.........................-------------
30241-swamp deer-Nepal         --------------.---..T.....---...........................------.........................-------------
30238-swamp deer-Nepal         --------------.---..T.....---...........................------.........................-------------
30243-swamp deer-Nepal         --------------.---..T.....---...........................------.........................-------------
32563-impala-S.Africa          --------------.---..T.....---...........................------.........................-------------
32560-buffalo-S.Africa         --------------.---..T.....---...........................------.........................-------------
31261-human-Turkey             --------------.---..T.....---...........................------.........................-------------
37828-human-UK                 --------------.---........---...........................------.........................-------------
37830-human-UK                 --------------.---........---...........................------.........................-------------
37827-human-UK                 --------------.---........---...........................------.........................-------------
31570-sheep-Spain              --------------.---..---...---T..........................------.........................-------------
37820-human-UK                 --------------.---..---...---T..........................------.........................-------------
```

```
37811-sheep-UK                  --------------.---........---T..........................------.........................-------------
37809-sheep-UK                  --------------.---........---T..........................------.........................-------------
37813-sheep-UK                  --------------.---........---T..........................------.........................-------------
37815-sheep-UK                  --------------.---........---T..........................------.........................-------------
37808-equine-UK                 --------------.---........---T..........................------.........................-------------
36629-sheep-Spain               --------------.---........---T..........................------.........................-------------
36628-sheep-Spain               --------------.---........---T..........................------.........................-------------
18165-sheep-Peru                --------------.---........---T..........................------.........................-------------
37821-human-UK                  --------------.---........---T..........................------.........................-------------
37818-human-UK                  --------------.---........---T..........................------.........................-------------
37825-human-UK                  --------------.---........---T..........................------.........................-------------
37829-human-UK                  --------------.---........---T..........................------.........................-------------
16421-human-Peru                --------------.---........---T..........................------.........................-------------
9870-human-Canada               --------------.---........---T..........................------.........................-------------
19716-alpaca-Peru               --------------.---........---T..........................------.........................-------------
35147-goat-Algeria              --------------.---........---T..........................------.........................-------------
18868-chipmunk-USA              --------------.---........---..C........................------............A........GA..-------------
33456-sifaka-USA                --------------.---........---..C........................------............A........GA..-------------
37822-human-UK                  --------------.---........---..C........................------............A........GA..-------------
37824-human-UK                  --------------.---........---..C........................------............A........GA..-------------
37819-human-UK                  --------------.---........---..C........................------............A........GA..-------------
37202-human-USA                 --------------.---........---..C........................------............A........GA..-------------
37192-human-USA                 --------------.---........---..C........................------............A........GA..-------------
37206-human-USA                 --------------.---........---..C........................------............A........GA..-------------
37191-human-USA                 --------------.---........---..C........................------............A........GA..-------------
34141-human-USA                 --------------.---........---..C........................------............A........GA..-------------
37215-human-USA                 --------------.---........---..C........................------............A........GA..-------------
37201-human-USA                 --------------.---........---..C........................------............A........GA..-------------
37212-human-USA                 --------------.---........---..C........................------............A........GA..-------------
29355-human-USA                 --------------.---........---..C........................------............A........GA..-------------
8512-storm-water-USA            --------------.---........---..C........................------............A........GA..-------------
8513-storm-water-USA            --------------.---........---..C........................------............A........GA..-------------
5397-storm-water-USA            --------------.---........---..C........................------............A........GA..-------------
6140-storm-water-USA            --------------.---........---..C........................------............A........GA..-------------
8509-storm-water-USA            --------------.---........---..C........................------............A........GA..-------------
8469-storm-water-USA            --------------.---........---..C........................------............A........GA..-------------
6141-storm-water-USA            --------------.---........---..C........................------............A........GA..-------------
8062-storm-water-USA            --------------.---........---..C........................------............A........GA..-------------
6143-storm-water-USA            --------------.---........---..C........................------............A........GA..-------------
6144-storm-water-USA            --------------.---........---..C........................------............A........GA..-------------
8514-storm-water-USA            --------------.---........---..C........................------............A........GA..-------------
8510-storm-water-USA            --------------.---........---..C........................------............A........GA..-------------
6317-storm-water-USA            --------------.---........---..C........................------............A........GA..-------------
7370-storm-water-USA            --------------.---........---..C........................------............A........GA..-------------
7163-storm-water-USA            --------------.---........---..C........................------............A........GA..-------------
15529-river-water-USA           --------------.---........---..C........................------............A........GA..-------------
14874-river-water-USA           --------------.---........---..C........................------............A........GA..-------------
18371-eastern gray squirrel-US  --------------.---........---..C---T....................------............A............-------------
18370-eastern gray squirrel-US  --------------.---........---..C---T....................------............A............-------------
14886-eastern gray squirrel-US  --------------.---........---..C---T....................------............A............-------------
14223-beaver-USA                --------------.---........---..C---T....................------............A............-------------
13266-red squirrel-USA          --------------.---........---..C---T....................------............A............-------------
37200-human-USA                 --------------.---........---..C---T....................------............A............-------------
37203-human-USA                 --------------.---........---..C---T....................------............A............-------------
37204-human-USA                 --------------.---........---..C---T....................------............A............-------------
37197-human-USA                 --------------.---........---..C---T....................------............A............-------------
37217-human-USA                 --------------.---........---..C---T....................------............A............-------------
37193-human-USA                 --------------.---........---..C---T....................------............A............-------------
29361-human-USA                 --------------.---........---..C---T....................------............A............-------------
8774-human-USA                  --------------.---........---..C---T....................------............A............-------------
37196-human-USA                 --------------.---........---..C---T....................------............A............-------------
37208-human-USA                 --------------.---........---..C---T....................------............A............-------------
37216-human-USA                 --------------.---........---..C---T....................------............A............-------------
37209-human-USA                 --------------.---........---..C---T....................------............A............-------------
37826-human-UK                  --------------.---........---..C---T....................------............A............-------------
37833-human-UK                  --------------.---........---..C---T....................------............A............-------------
22508-river-water-USA           --------------.---........---..C---T....................------............A............-------------
13264-woodchuck-USA             --------------.---........---..C---T....................------............A............-------------
12963-raccoon-USA               --------------.---........---..C---T....................------............A............-------------
36641-porcupine-USA             --------------.---........---..C........................------.....................GA..-------------
36642-porcupine-USA             --------------.---........---..C........................------.....................GA..-------------
36638-porcupine via goat-USA    --------------.---........---..C........................------.....................GA..-------------
36639-porcupine via goat-USA    --------------.---........---..C........................------.....................GA..-------------
36640-porcupine via goat-USA    --------------.---........---..C........................------.....................GA..-------------
17654-human-USA                 --------------.---........---..C........................------.....................GA..-------------
17577-human-USA                 --------------.---........---..C........................------.....................GA..-------------
37211-human-USA                 --------------.---........---..C........................------.....................GA..-------------
17574-human-USA                 --------------.---........---..C........................------.....................GA..-------------
37640-field mouse-Slovak Repub  CAGCAACAACTCCT.GTG..A.....CCT.G....A.T...C...G.GC......TCCTGGT...A..A..CCT..T.C.AC.....CTGGTGCTACTAC
37642-field mouse-Slovak Repub  CAGCAACAACTCCT.GTG..A.....CCT.G....A.T...C...G.GC......TCCTGGT...A..A..CCT..T.C.AC.....CTGGTGCTACTAC
37644-field mouse-Slovak Repub  CAGCAACAACTCCT.GTG..A.....CCT.G....A.T...C...G.GC......TCCTGGT...A..A..CCT..T.C.AC.....CTGGTGCTACTAC
37646-field mouse-Slovak Repub  --------------.GTGT.A.C...---.T......TGTGG.A.G.GT...C..T------......GTG.CA..T.T.ACC...------GCTCCTGT
AF114166-C. parvum-gp60 gene    --------------.GAGAAGACG.AGAA.GCAG..AAGA.T..AG.G....GAAG----------.......TA..CAG.G.T..GAAGAGGAA-----
Clustal Consensus                             *         *                *    *  ***              **       *         *

                                       410       420       430       440       450       460       470       480       490       500
                                ....|....|....|....|....|....|....|....|....|....|....|....|....|....|....|....|....|....|....|....|
36625-sheep-Brazil              -----------------------------C------------TA---------------------GCACTGAT------------GGAGATGAGACT---
36624-sheep-Brazil              -----------------------------.------------..---------------------........------------............---
36623-sheep-Brazil              -----------------------------.------------..---------------------........------------............---
36630-sheep-Spain               -----------------------------.------------..---------------------........------------............---
36631-sheep-Spain               -----------------------------.------------..---------------------........------------............---
36633-sheep-Spain               -----------------------------.------------..---------------------........------------............---
36627-sheep-Spain               -----------------------------.------------..---------------------........------------............---
36632-sheep-Spain               -----------------------------.------------..---------------------........------------............---
36626-sheep-Spain               -----------------------------.------------..---------------------........------------............---
36634-sheep-USA                 -----------------------------.------------..---------------------........------------............---
36637-sheep-USA                 -----------------------------.------------..---------------------........------------............---
37766-sheep-USA                 -----------------------------.------------..---------------------........------------............---
36636-sheep-USA                 -----------------------------.------------..---------------------........------------............---
37769-sheep-USA                 -----------------------------.------------..---------------------........------------............---
37767-sheep-USA                 -----------------------------.------------..---------------------........------------............---
37768-sheep-USA                 -----------------------------.------------..---------------------........------------............---
```

```
37774-sheep-USA                 -----------------------------.------------..---------------------........------------............---
37777-sheep-USA                 -----------------------------.------------..---------------------........------------............---
37776-sheep-USA                 -----------------------------.------------..---------------------........------------............---
37775-sheep-USA                 -----------------------------.------------..---------------------........------------............---
37772-sheep-USA                 -----------------------------.------------..---------------------........------------............---
37773-sheep-USA                 -----------------------------.------------..---------------------........------------............---
37771-sheep-USA                 -----------------------------.------------..---------------------........------------............---
6478-blesbok-Czech Republic     -----------------------------.------------..CT------------------A........------------............---
6482-nyala-Czech Republic       -----------------------------.------------..CT------------------A........------------............---
34023-sheep-China               -----------------------------.------------..CT------------------A........------------............---
36737-sheep-China               -----------------------------.------------..CT------------------A........------------............---
34022-sheep-China               -----------------------------.------------..CT------------------A........------------............---
38161-sheep-Australia           -----------------------------.------------..CT------------------A........------------............---
38157-sheep-Australia           -----------------------------.------------..CT------------------A........------------............---
38156-sheep-Australia           -----------------------------.------------..CT------------------A........------------............---
38154-sheep-Australia           -----------------------------.------------..CT------------------A........------------............---
38153-sheep-Australia           -----------------------------.------------..CT------------------A........------------............---
38152-sheep-Australia           -----------------------------.------------..CT------------------A........------------............---
34049-sheep-China               -----------------------------.------------..CT------------------A........------------............---
37635-sheep-Turkey              -----------------------------.------------..---------------------........------------............---
34043-sheep-China               -----------------------------.------------..CT------------------A........------------............---
34047-sheep-China               -----------------------------.------------..CT------------------A........------------............---
34055-sheep-China               -----------------------------.------------..CT------------------A........------------............---
34011-sheep-China               -----------------------------.------------..CT------------------A........------------............---
34038-sheep-China               -----------------------------.------------..CT------------------A........------------............---
34044-sheep-China               -----------------------------.------------..CT------------------A........------------............---
34032-sheep-China               -----------------------------.------------..CT------------------A........------------............---
34060-sheep-China               -----------------------------.------------..CT------------------A........------------............---
34048-sheep-China               -----------------------------.------------..CT------------------A........------------............---
36745-sheep-China               -----------------------------.------------..CT------------------A........------------............---
36785-sheep-China               -----------------------------.------------..CT------------------A........------------............---
34025-sheep-China               -----------------------------.------------..CT------------------A........------------............---
34034-sheep-China               -----------------------------.------------..CT------------------A........------------............---
19141-yak-China                 -----------------------------.------------..CT------------------A........------------............---
19142-yak-China                 -----------------------------.------------..CT------------------A........------------............---
28682-yak-China                 -----------------------------.------------..CT------------------A........------------............---
30241-swamp deer-Nepal          -----------------------------.------------..CT------------------A........------------............---
30238-swamp deer-Nepal          -----------------------------.------------..CT------------------A........------------............---
30243-swamp deer-Nepal          -----------------------------.------------..CT------------------A........------------............---
32563-impala-S.Africa           -----------------------------.------------..CT------------------A........------------............---
32560-buffallo-S.Africa         -----------------------------.------------..CT------------------A........------------............---
31261-human-Turkey              -----------------------------.------------..CT------------------A........------------............---
37828-human-UK                  -----------------------------.------------..CT------------------A........------------............---
37830-human-UK                  -----------------------------.------------..CT------------------A........------------............---
37827-human-UK                  -----------------------------.------------..CT------------------A........------------............---
31570-sheep-Spain               -----------------------------.------------..CT------------------A........------------............---
37820-human-UK                  -----------------------------.------------..CT------------------A........------------............---
37811-sheep-UK                  -----------------------------.------------..CT------------------A........------------............---
37809-sheep-UK                  -----------------------------.------------..CT------------------A........------------............---
37813-sheep-UK                  -----------------------------.------------..CT------------------A........------------............---
37815-sheep-UK                  -----------------------------.------------..CT------------------A........------------............---
37808-equine-UK                 -----------------------------.------------..CT------------------A........------------............---
36629-sheep-Spain               -----------------------------.------------..CT------------------A........------------............---
36628-sheep-Spain               -----------------------------.------------..CT------------------A........------------............---
18165-sheep-Peru                -----------------------------.------------..CT------------------A........------------............---
37821-human-UK                  -----------------------------.------------..CT------------------A........------------............---
37818-human-UK                  -----------------------------.------------..CT------------------A........------------............---
37825-human-UK                  -----------------------------.------------..CT------------------A........------------............---
37829-human-UK                  -----------------------------.------------..CT------------------A........------------............---
16421-human-Peru                -----------------------------.------------..CT------------------A........------------............---
9870-human-Canada               -----------------------------.------------..CT------------------A........------------............---
19716-alpaca-Peru               -----------------------------.------------..CT------------------A........------------............---
35147-goat-Algeria              -----------------------------.------------..CT------------------A........------------............---
18868-chipmunk-USA              -----------------------------.------------..CT------------------A........GGA---------.AT..GA.....GAT
33456-sifaka-USA                -----------------------------.------------..CT------------------A........GGA---------.AT..GA.....GAT
37822-human-UK                  -----------------------------.------------..CT------------------A........GGA---------.AT..GA.....GAT
37824-human-UK                  -----------------------------.------------..CT------------------A........GGA---------.AT..GA.....GAT
37819-human-UK                  -----------------------------.------------..CT------------------A........GGA---------.AT..GA.....GAT
37202-human-USA                 -----------------------------.------------..CT------------------A........GGA---------.AT..GA.....GAT
37192-human-USA                 -----------------------------.------------..CT------------------A........GGA---------.AT..GA.....GAT
37206-human-USA                 -----------------------------.------------..CT------------------A........GGA---------.AT..GA.....GAT
37191-human-USA                 -----------------------------.------------..CT------------------A........GGA---------.AT..GA.....GAT
34141-human-USA                 -----------------------------.------------..CT------------------A........GGA---------.AT..GA.....GAT
37215-human-USA                 -----------------------------.------------..CT------------------A........GGA---------.AT..GA.....GAT
37201-human-USA                 -----------------------------.------------..CT------------------A........GGA---------.AT..GA.....GAT
37212-human-USA                 -----------------------------.------------..CT------------------A........GGA---------.AT..GA.....GAT
29355-human-USA                 -----------------------------.------------..CT------------------A........GGA---------.AT..GA.....GAT
8512-storm-water-USA            -----------------------------.------------..CT------------------A........GGA---------.AT..GA.....GAT
8513-storm-water-USA            -----------------------------.------------..CT------------------A........GGA---------.AT..GA.....GAT
5397-storm-water-USA            -----------------------------.------------..CT------------------A........GGA---------.AT..GA.....GAT
6140-storm-water-USA            -----------------------------.------------..CT------------------A........GGA---------.AT..GA.....GAT
8509-storm-water-USA            -----------------------------.------------..CT------------------A........GGA---------.AT..GA.....GAT
8469-storm-water-USA            -----------------------------.------------..CT------------------A........GGA---------.AT..GA.....GAT
6141-storm-water-USA            -----------------------------.------------..CT------------------A........GGA---------.AT..GA.....GAT
8062-storm-water-USA            -----------------------------.------------..CT------------------A........GGA---------.AT..GA.....GAT
6143-storm-water-USA            -----------------------------.------------..CT------------------A........GGA---------.AT..GA.....GAT
6144-storm-water-USA            -----------------------------.------------..CT------------------A........GGA---------.AT..GA.....GAT
8514-storm-water-USA            -----------------------------.------------..CT------------------A........GGA---------.AT..GA.....GAT
8510-storm-water-USA            -----------------------------.------------..CT------------------A........GGA---------.AT..GA.....GAT
6317-storm-water-USA            -----------------------------.------------..CT------------------A........GGA---------.AT..GA.....GAT
7370-storm-water-USA            -----------------------------.------------..CT------------------A........GGA---------.AT..GA.....GAT
7163-storm-water-USA            -----------------------------.------------..CT------------------A........GGA---------.AT..GA.....GAT
15529-river-water-USA           -----------------------------.------------..CT------------------A........GGA---------.AT..GA.....GAT
14874-river-water-USA           -----------------------------.------------..CT------------------A........GGA---------.AT..GA.....GAT
18371-eastern gray squirrel-US  -----------------------------.------------..CT------------------A........GGAGAG------.AT..GA.....GAT
18370-eastern gray squirrel-US  -----------------------------.------------..CT------------------A........GGAGAG------.AT..GA.....GAT
14886-eastern gray squirrel-US  -----------------------------.------------..CT------------------A........GGAGAG------.AT..GA.....GAT
14223-beaver-USA                -----------------------------.------------..CT------------------A........GGAGAG------.AT..GA.....GAT
13266-red squirrel-USA          -----------------------------.------------..CT------------------A........GGAGAG------.AT..GA.....GAT
37200-human-USA                 -----------------------------.------------..CT------------------A........GGAGAG------.AT..GA.....GAT
37203-human-USA                 -----------------------------.------------..CT------------------A........GGAGAG------.AT..GA.....GAT
37204-human-USA                 -----------------------------.------------..CT------------------A........GGAGAG------.AT..GA.....GAT
37197-human-USA                 -----------------------------.------------..CT------------------A........GGAGAG------.AT..GA.....GAT
```

```
37217-human-USA                 -----------------------------.------------..CT------------------A........GGAGAG------.AT..GA.....GAT
37193-human-USA                 -----------------------------.------------..CT------------------A........GGAGAG------.AT..GA.....GAT
29361-human-USA                 -----------------------------.------------..CT------------------A........GGAGAG------.AT..GA.....GAT
8774-human-USA                  -----------------------------.------------..CT------------------A........GGAGAG------.AT..GA.....GAT
37196-human-USA                 -----------------------------.------------..CT------------------A........GGAGAG------.AT..GA.....GAT
37208-human-USA                 -----------------------------.------------..CT------------------A........GGAGAG------.AT..GA.....GAT
37216-human-USA                 -----------------------------.------------..CT------------------A........GGAGAG------.AT..GA.....GAT
37209-human-USA                 -----------------------------.------------..CT------------------A........GGAGAG------.AT..GA.....GAT
37826-human-UK                  -----------------------------.------------..CT------------------A........GGAGAG------.AT..GA.....GAT
37833-human-UK                  -----------------------------.------------..CT------------------A........GGAGAG------.AT..GA.....GAT
22508-river-water-USA           -----------------------------.------------..CT------------------A........GGAGAG------.AT..GA.....GAT
13264-woodchuck-USA             -----------------------------.------------..CT------------------A........GGAGAG------.AT..GA.....GAT
12963-raccoon-USA               -----------------------------.------------..CT------------------A........GGAGAG------.AT..GA.....GAT
36641-porcupine-USA             -----------------------------.------------..CT------------------A........GGA---------.AT..GA.....GAT
36642-porcupine-USA             -----------------------------.------------..CT------------------A........GGA---------.AT..GA.....GAT
36638-porcupine via goat-USA    -----------------------------.------------..CT------------------A........GGA---------.AT..GA.....GAT
36639-porcupine via goat-USA    -----------------------------.------------..CT------------------A........GGA---------.AT..GA.....GAT
36640-porcupine via goat-USA    -----------------------------.------------..CT------------------A........GGA---------.AT..GA.....GAT
17654-human-USA                 -----------------------------.------------..CT------------------A........GGA---------.AT..GA.....GAT
17577-human-USA                 -----------------------------.------------..CT------------------A........GGA---------.AT..GA.....GAT
37211-human-USA                 -----------------------------.------------..CT------------------A........GGA---------.AT..GA.....GAT
17574-human-USA                 -----------------------------.------------..CT------------------A........GGA---------.AT..GA.....GAT
37640-field mouse-Slovak Repub  TCCTGGTGCTACTACTCCTGGTGCTACTA.TCCTGGTGCTAC..CTCCTGGTGCTACTACTGGTACT....G.ACTACTGGAACA.TT....GTT.CGGG
37642-field mouse-Slovak Repub  TCCTGGTGCTACTACTCCTGGTGCTACTA.TCCTGGTGCTAC..CTCCTGGTGCTACTACTGGTACT....G.ACTACTGGAACA.TT....GTT.CGGG
37644-field mouse-Slovak Repub  TCCTGGTGCTACTACTCCTGGTGCTACTA.TCCTGGTGCTAC..CTCCTGGTGCTACTACTGGTACT....G.ACTACTGGAACA.TT....GTT.CGGG
37646-field mouse-Slovak Repub  TACTGGTGTTACCACTGTTG---------.TCCAGGTGTTCCC.CT------------------ACAG.C...------------.TT....GC...GGA
AF114166-C. parvum-gp60 gene    -------------------------------GGTAGTGAAGACGAT---------------GGCCAA...AG.GCT---------.CTTCCC.AC.CACT
Clustal Consensus                                                                                   *   *            *         *

                                       510       520       530       540       550       560       570       580       590       600
                                ....|....|....|....|....|....|....|....|....|....|....|....|....|....|....|....|....|....|....|....|
36625-sheep-Brazil              ACTGACAC---TGGTAGTGGTACTAC------CGGCGAAACAGTTACAACTACTCCTGACCCAATGGAGAAATGCGGTCTTTCATTCGTCATGTGGTTTG
36624-sheep-Brazil              ........---...............------....................................................................
36623-sheep-Brazil              ........---...............------....................................................................
36630-sheep-Spain               ........---...............------....................................................................
36631-sheep-Spain               ........---...............------....................................................................
36633-sheep-Spain               ........---...............------....................................................................
36627-sheep-Spain               ........---...............------....................................................................
36632-sheep-Spain               ........---...............------....................................................................
36626-sheep-Spain               ........---...............------....................................................................
36634-sheep-USA                 ........---...............------....................................................................
36637-sheep-USA                 ........---...............------....................................................................
37766-sheep-USA                 ........---...............------....................................................................
36636-sheep-USA                 ........---...............------....................................................................
37769-sheep-USA                 ........---...............------....................................................................
37767-sheep-USA                 ........---...............------....................................................................
37768-sheep-USA                 ........---...............------....................................................................
37774-sheep-USA                 ........---...............------....................................................................
37777-sheep-USA                 ........---...............------....................................................................
37776-sheep-USA                 ........---...............------....................................................................
37775-sheep-USA                 ........---...............------....................................................................
37772-sheep-USA                 ........---...............------....................................................................
37773-sheep-USA                 ........---...............------....................................................................
37771-sheep-USA                 ........---...............------....................................................................
6478-blesbok-Czech Republic     ........---...............------....................................................................
6482-nyala-Czech Republic       ........---...............------....................................................................
34023-sheep-China               ........---...............------....................................................................
36737-sheep-China               ........---...............------....................................................................
34022-sheep-China               ........---...............------....................................................................
38161-sheep-Australia           ........---...............------....................................................................
38157-sheep-Australia           ........---...............------....................................................................
38156-sheep-Australia           ........---...............------....................................................................
38154-sheep-Australia           ........---...............------....................................................................
38153-sheep-Australia           ........---...............------....................................................................
38152-sheep-Australia           ........---...............------....................................................................
34049-sheep-China               ........---...............------....................................................................
37635-sheep-Turkey              ........---...............------....................................................................
34043-sheep-China               ........---...............------....................................................................
34047-sheep-China               ........---...............------....................................................................
34055-sheep-China               ........---...............------....................................................................
34011-sheep-China               ........---...............------....................................................................
34038-sheep-China               ........---...............------....................................................................
34044-sheep-China               ........---...............------....................................................................
34032-sheep-China               ........---...............------....................................................................
34060-sheep-China               ........---...............------....................................................................
34048-sheep-China               ........---...............------....................................................................
36745-sheep-China               ........---...............------....................................................................
36785-sheep-China               ........---...............------....................................................................
34025-sheep-China               ........---...............------....................................................................
34034-sheep-China               ........---...............------....................................................................
19141-yak-China                 ........---...............------....................................................................
19142-yak-China                 ........---...............------....................................................................
28682-yak-China                 ........---...............------....................................................................
30241-swamp deer-Nepal          ........---...............------....................................................................
30238-swamp deer-Nepal          ........---...............------....................................................................
30243-swamp deer-Nepal          ........---...............------....................................................................
32563-impala-S.Africa           ........---...............------....................................................................
32560-buffallo-S.Africa         ........---...............------....................................................................
31261-human-Turkey              ........---...............------....................................................................
37828-human-UK                  ........---..A............------....................................................................
37830-human-UK                  ........---..A............------....................................................................
37827-human-UK                  ........---..A............------....................................................................
31570-sheep-Spain               ........---...............------....................................................................
37820-human-UK                  ........---...............------....................................................................
37811-sheep-UK                  ........---...............------....................................................................
37809-sheep-UK                  ........---...............------....................................................................
37813-sheep-UK                  ........---...............------....................................................................
37815-sheep-UK                  ........---...............------....................................................................
37808-equine-UK                 ........---...............------....................................................................
36629-sheep-Spain               ........---...............------....................................................................
36628-sheep-Spain               ........---...............------....................................................................
18165-sheep-Peru                ........---...............------....................................................................
37821-human-UK                  ........---...............------....................................................................
```

```
37818-human-UK                    ........---...............------....................................................................
37825-human-UK                    ........---...............------....................................................................
37829-human-UK                    ........---...............------....................................................................
16421-human-Peru                  ........---...............------....................................................................
9870-human-Canada                 ........---...............------....................................................................
19716-alpaca-Peru                 ........---...............------....................................................................
35147-goat-Algeria                ........---...............------....................................................................
18868-chipmunk-USA                ........---...............------..A...........................................AC....................
33456-sifaka-USA                  ........---...............------..A...........................................AC....................
37822-human-UK                    ........---...............------..A...........................................AC....................
37824-human-UK                    ........---...............------..A...........................................AC....................
37819-human-UK                    ........---...............------..A...........................................AC....................
37202-human-USA                   ........---...............------..A...........................................AC....................
37192-human-USA                   ........---...............------..A...........................................AC....................
37206-human-USA                   ........---...............------..A...........................................AC....................
37191-human-USA                   ........---...............------..A...........................................AC....................
34141-human-USA                   ........---...............------..A...........................................AC....................
37215-human-USA                   ........---...............------..A...........................................AC....................
37201-human-USA                   ........---...............------..A...........................................AC....................
37212-human-USA                   ........---...............------..A...........................................AC....................
29355-human-USA                   ........---...............------..A...........................................AC....................
8512-storm-water-USA              ........---...............------..A...........................................AC....................
8513-storm-water-USA              ........---...............------..A...........................................AC....................
5397-storm-water-USA              ........---...............------..A...........................................AC....................
6140-storm-water-USA              ........---...............------..A...........................................AC....................
8509-storm-water-USA              ........---...............------..A...........................................AC....................
8469-storm-water-USA              ........---...............------..A...........................................AC....................
6141-storm-water-USA              ........---...............------..A...........................................AC....................
8062-storm-water-USA              ........---...............------..A...........................................AC....................
6143-storm-water-USA              ........---...............------..A...........................................AC....................
6144-storm-water-USA              ........---...............------..A...........................................AC....................
8514-storm-water-USA              ........---...............------..A...........................................AC....................
8510-storm-water-USA              ........---...............------..A...........................................AC....................
6317-storm-water-USA              ........---...............------..A...........................................AC....................
7370-storm-water-USA              ........---...............------..A...........................................AC....................
7163-storm-water-USA              ........---...............------..A...........................................AC....................
15529-river-water-USA             ........---...............------..A...........................................AC....................
14874-river-water-USA             ........---...............------..A...........................................AC....................
18371-eastern gray squirrel-US    ........TGA...............------..AT................................................................
18370-eastern gray squirrel-US    ........TGA...............------..AT................................................................
14886-eastern gray squirrel-US    ........TGA...............------..AT................................................................
14223-beaver-USA                  ........TGA...............------..AT................................................................
13266-red squirrel-USA            ........TGA...............------..AT................................................................
37200-human-USA                   ........TGA...............------..AT................................................................
37203-human-USA                   ........TGA...............------..AT................................................................
37204-human-USA                   ........TGA...............------..AT................................................................
37197-human-USA                   ........TGA...............------..AT................................................................
37217-human-USA                   ........TGA...............------..AT................................................................
37193-human-USA                   ........TGA...............------..AT................................................................
29361-human-USA                   ........TGA...............------..AT................................................................
8774-human-USA                    ........TGA...............------..AT................................................................
37196-human-USA                   ........TGA...............------..AT................................................................
37208-human-USA                   ........TGA...............------..AT................................................................
37216-human-USA                   ........TGA...............------..AT................................................................
37209-human-USA                   ........TGA...............------..AT................................................................
37826-human-UK                    ........TGA...............------..AT................................................................
37833-human-UK                    ........TGA...............------..AT................................................................
22508-river-water-USA             ........TGA...............------..AT................................................................
13264-woodchuck-USA               ........TGA...............------..AT................................................................
12963-raccoon-USA                 ........TGA...............------..AT................................................................
36641-porcupine-USA               ........---...............------..A......................................................T..........
36642-porcupine-USA               ........---...............------..A......................................................T..........
36638-porcupine via goat-USA      ........---...............------..A......................................................T..........
36639-porcupine via goat-USA      ........---...............------..A......................................................T..........
36640-porcupine via goat-USA      ........---...............------..A......................................................T..........
17654-human-USA                   ........---...............------..A......................................................T..........
17577-human-USA                   ........---...............------..A......................................................T..........
37211-human-USA                   ........---...............------..A......................................................T..........
17574-human-USA                   ........---...............------..A......................................................T..........
37640-field mouse-Slovak Repub    GA...A------....C...AGA.GA------T.CT.GT..T.CA...........A......GCC...C.G.................T..........
37642-field mouse-Slovak Repub    GA...A------....C...AGA.GA------T.CT.GT..T.CA...........A......GCC...C.G.................T..........
37644-field mouse-Slovak Repub    GA...A------....C...AGA.GA------T.CT.GT..T.CA...........A......GCC...C.G.................T..........
37646-field mouse-Slovak Repub    GGA..G------...GA.ACA.G...------T..T..G..T.GG...GT......C.......CT........T...........T..T..........
AF114166-C. parvum-gp60 gene      ...CCAG.TCAAA..GAA..CG.A..------TAC......CA.AGA.G.......------..AA..AG.......CAC......T..A........C.
Clustal Consensus                               *                     *  **      *  ******      *   **  * ** **   ****** ** ******** *

                                          610       620       630       640       650       660       670       680       690       700
                                  ....|....|....|....|....|....|....|....|....|....|....|....|....|....|....|....|....|....|....|....|
36625-sheep-Brazil                TTAGTGGTACTCCAGTTACAACTCTCGAATGTGGACCTTACACCATGGTATATGGACCAGTTGAAGGGGAGACTAACCCTGCAGCAAGGTATGTCTCTGG
36624-sheep-Brazil                ....................................................................................................
36623-sheep-Brazil                ....................................................................................................
36630-sheep-Spain                 ....................................................................................................
36631-sheep-Spain                 ....................................................................................................
36633-sheep-Spain                 ....................................................................................................
36627-sheep-Spain                 ....................................................................................................
36632-sheep-Spain                 ....................................................................................................
36626-sheep-Spain                 ....................................................................................................
36634-sheep-USA                   ....................................................................................................
36637-sheep-USA                   ....................................................................................................
37766-sheep-USA                   ....................................................................................................
36636-sheep-USA                   ....................................................................................................
37769-sheep-USA                   ....................................................................................................
37767-sheep-USA                   ....................................................................................................
37768-sheep-USA                   ....................................................................................................
37774-sheep-USA                   ....................................................................................................
37777-sheep-USA                   ....................................................................................................
37776-sheep-USA                   ....................................................................................................
37775-sheep-USA                   ....................................................................................................
37772-sheep-USA                   ....................................................................................................
37773-sheep-USA                   ....................................................................................................
37771-sheep-USA                   ....................................................................................................
6478-blesbok-Czech Republic       ....................................................................................................
6482-nyala-Czech Republic         ....................................................................................................
```

```
34023-sheep-China               ....................................................................................................
36737-sheep-China               ....................................................................................................
34022-sheep-China               ....................................................................................................
38161-sheep-Australia           ....................................................................................................
38157-sheep-Australia           ....................................................................................................
38156-sheep-Australia           ....................................................................................................
38154-sheep-Australia           ....................................................................................................
38153-sheep-Australia           ....................................................................................................
38152-sheep-Australia           ....................................................................................................
34049-sheep-China               ....................................................................................................
37635-sheep-Turkey              ....................................................................................................
34043-sheep-China               ....................................................................................................
34047-sheep-China               ....................................................................................................
34055-sheep-China               ....................................................................................................
34011-sheep-China               ....................................................................................................
34038-sheep-China               ....................................................................................................
34044-sheep-China               ....................................................................................................
34032-sheep-China               ....................................................................................................
34060-sheep-China               ....................................................................................................
34048-sheep-China               ....................................................................................................
36745-sheep-China               ....................................................................................................
36785-sheep-China               ....................................................................................................
34025-sheep-China               ....................................................................................................
34034-sheep-China               ....................................................................................................
19141-yak-China                 ....................................................................................................
19142-yak-China                 ....................................................................................................
28682-yak-China                 ....................................................................................................
30241-swamp deer-Nepal          ....................................................................................................
30238-swamp deer-Nepal          ....................................................................................................
30243-swamp deer-Nepal          ....................................................................................................
32563-impala-S.Africa           ....................................................................................................
32560-buffallo-S.Africa         ....................................................................................................
31261-human-Turkey              ....................................................................................................
37828-human-UK                  ....................................................................................................
37830-human-UK                  ....................................................................................................
37827-human-UK                  ....................................................................................................
31570-sheep-Spain               ....................................................................................................
37820-human-UK                  ....................................................................................................
37811-sheep-UK                  ....................................................................................................
37809-sheep-UK                  ....................................................................................................
37813-sheep-UK                  ....................................................................................................
37815-sheep-UK                  ....................................................................................................
37808-equine-UK                 ....................................................................................................
36629-sheep-Spain               ....................................................................................................
36628-sheep-Spain               ....................................................................................................
18165-sheep-Peru                ....................................................................................................
37821-human-UK                  ....................................................................................................
37818-human-UK                  ....................................................................................................
37825-human-UK                  ....................................................................................................
37829-human-UK                  ....................................................................................................
16421-human-Peru                ....................................................................................................
9870-human-Canada               ....................................................................................................
19716-alpaca-Peru               ....................................................................................................
35147-goat-Algeria              ....................................................................................................
18868-chipmunk-USA              .......CGT.........................T................C...........GAAT................................
33456-sifaka-USA                .......CGT.........................T................C...........GAAT................................
37822-human-UK                  .......CGT.........................T................C...........GAAT................................
37824-human-UK                  .......CGT.........................T................C...........GAAT................................
37819-human-UK                  .......CGT.........................T................C...........GAAT................................
37202-human-USA                 .......CGT.........................T................C...........GAAT................................
37192-human-USA                 .......CGT.........................T................C...........GAAT................................
37206-human-USA                 .......CGT.........................T................C...........GAAT................................
37191-human-USA                 .......CGT.........................T................C...........GAAT................................
34141-human-USA                 .......CGT.........................T................C...........GAAT................................
37215-human-USA                 .......CGT.........................T................C...........GAAT................................
37201-human-USA                 .......CGT.........................T................C...........GAAT................................
37212-human-USA                 .......CGT.........................T................C...........GAAT................................
29355-human-USA                 .......CGT.........................T................C...........GAAT................................
8512-storm-water-USA            .......CGT.........................T................C...........GAAT................................
8513-storm-water-USA            .......CGT.........................T................C...........GAAT................................
5397-storm-water-USA            .......CGT.........................T................C...........GAAT................................
6140-storm-water-USA            .......CGT.........................T................C...........GAAT................................
8509-storm-water-USA            .......CGT.........................T................C...........GAAT................................
8469-storm-water-USA            .......CGT.........................T................C...........GAAT................................
6141-storm-water-USA            .......CGT.........................T................C...........GAAT................................
8062-storm-water-USA            .......CGT.........................T................C...........GAAT................................
6143-storm-water-USA            .......CGT.........................T................C...........GAAT................................
6144-storm-water-USA            .......CGT.........................T................C...........GAAT................................
8514-storm-water-USA            .......CGT.........................T................C...........GAAT................................
8510-storm-water-USA            .......CGT.........................T................C...........GAAT................................
6317-storm-water-USA            .......CGT.........................T................C...........GAAT................................
7370-storm-water-USA            .......CGT.........................T................C...........GAAT................................
7163-storm-water-USA            .......CGT.........................T................C...........GAAT................................
15529-river-water-USA           .......CGT.........................T................C...........GAAT................................
14874-river-water-USA           .......CGT.........................T................C...........GAAT................................
18371-eastern gray squirrel-US  ......................C.....G....................T..C..G...A....T...................................
18370-eastern gray squirrel-US  ......................C.....G....................T..C..G...A....T...................................
14886-eastern gray squirrel-US  ......................C.....G....................T..C..G...A....T...................................
14223-beaver-USA                ......................C.....G....................T..C..G...A....T...................................
13266-red squirrel-USA          ......................C.....G....................T..C..G...A....T...................................
37200-human-USA                 ......................C.....G....................T..C..G...A....T...................................
37203-human-USA                 ......................C.....G....................T..C..G...A....T...................................
37204-human-USA                 ......................C.....G....................T..C..G...A....T...................................
37197-human-USA                 ......................C.....G....................T..C..G...A....T...................................
37217-human-USA                 ......................C.....G....................T..C..G...A....T...................................
37193-human-USA                 ......................C.....G....................T..C..G...A....T...................................
29361-human-USA                 ......................C.....G....................T..C..G...A....T...................................
8774-human-USA                  ......................C.....G....................T..C..G...A....T...................................
37196-human-USA                 ......................C.....G....................T..C..G...A....T...................................
37208-human-USA                 ......................C.....G....................T..C..G...A....T...................................
37216-human-USA                 ......................C.....G....................T..C..G...A....T...................................
37209-human-USA                 ......................C.....G....................T..C..G...A....T...................................
37826-human-UK                  ......................C.....G....................T..C..G...A....T...................................
```

```
37833-human-UK                      ......................C.....G....................T..C..G...A....T...................................
22508-river-water-USA               ......................C.....G....................T..C..G...A....T...................................
13264-woodchuck-USA                 ......................C.....G....................T..C..G...A....T...................................
12963-raccoon-USA                   ......................C.....G....................T..C..G...A....T...................................
36641-porcupine-USA                 ............................G....................T..C..G...A....T...................................
36642-porcupine-USA                 ............................G....................T..C..G...A....T...................................
36638-porcupine via goat-USA        ............................G....................T..C..G...A....T...................................
36639-porcupine via goat-USA        ............................G....................T..C..G...A....T...................................
36640-porcupine via goat-USA        ............................G....................T..C..G...A....T...................................
17654-human-USA                     ............................G....................T..C..G...A....T...................................
17577-human-USA                     ............................G....................T..C..G...A....T...................................
37211-human-USA                     ............................G....................T..C..G...A....T...................................
17574-human-USA                     ............................G....................T..C..G...A....T...................................
37640-field mouse-Slovak Repub      ..G.....GT.........G........C......G..........................A.G..T..T.G......A..............G.....
37642-field mouse-Slovak Repub      ..G.....GT.........G........C......G..........................A.G..T..T.G......A..............G.....
37644-field mouse-Slovak Repub      ..G.....GT.........G........C......G..........................A.G..T..T.G......A..............G.....
37646-field mouse-Slovak Repub      .......AGT..................G......G..........T.....C..G........G..T..A........G........A...........
AF114166-C. parvum-gp60 gene        GAGAA.....C....C.G.G..AT.GA.G.....TG.C.....T..C..C....C...TA.AA...ACC.A..AG.T..C...C....A...A.......
Clustal Consensus                        **    **** * * **  *  * *****  * ***** ** ** ** *  **  *  *     * *   * ** *** **** *** * *****

                                             710       720       730       740       750       760       770       780       790       800
                                    ....|....|....|....|....|....|....|....|....|....|....|....|....|....|....|....|....|....|....|....|
36625-sheep-Brazil                  GCCCGTTACTACGGTTACTTATGAAGAAAGCAGCAAGA------AACTCATGATCAATGGCCAGGAATTCGCCACTCTTTCCATGGATTCATCAAAACCA
36624-sheep-Brazil                  ......................................------........................................................
36623-sheep-Brazil                  ......................................------........................................................
36630-sheep-Spain                   ......................................------........................................................
36631-sheep-Spain                   ......................................------........................................................
36633-sheep-Spain                   ......................................------........................................................
36627-sheep-Spain                   ......................................------........................................................
36632-sheep-Spain                   ......................................------........................................................
36626-sheep-Spain                   ......................................------........................................................
36634-sheep-USA                     ......................................------........................................................
36637-sheep-USA                     ......................................------........................................................
37766-sheep-USA                     ......................................------........................................................
36636-sheep-USA                     ......................................------........................................................
37769-sheep-USA                     ......................................------........................................................
37767-sheep-USA                     ......................................------........................................................
37768-sheep-USA                     ......................................------........................................................
37774-sheep-USA                     ......................................------........................................................
37777-sheep-USA                     ......................................------........................................................
37776-sheep-USA                     ......................................------........................................................
37775-sheep-USA                     ......................................------........................................................
37772-sheep-USA                     ......................................------........................................................
37773-sheep-USA                     ......................................------........................................................
37771-sheep-USA                     ......................................------........................................................
6478-blesbok-Czech Republic         ......................................------........................................................
6482-nyala-Czech Republic           ......................................------........................................................
34023-sheep-China                   ......................................------........................................................
36737-sheep-China                   ......................................------........................................................
34022-sheep-China                   ......................................------........................................................
38161-sheep-Australia               ......................................------........................................................
38157-sheep-Australia               ......................................------........................................................
38156-sheep-Australia               ......................................------........................................................
38154-sheep-Australia               ......................................------........................................................
38153-sheep-Australia               ......................................------........................................................
38152-sheep-Australia               ......................................------........................................................
34049-sheep-China                   ......................................------........................................................
37635-sheep-Turkey                  ......................................------........................................................
34043-sheep-China                   ......................................------........................................................
34047-sheep-China                   ......................................------........................................................
34055-sheep-China                   ......................................------........................................................
34011-sheep-China                   ......................................------........................................................
34038-sheep-China                   ......................................------........................................................
34044-sheep-China                   ......................................------........................................................
34032-sheep-China                   ......................................------........................................................
34060-sheep-China                   ......................................------........................................................
34048-sheep-China                   ......................................------........................................................
36745-sheep-China                   ......................................------........................................................
36785-sheep-China                   ......................................------........................................................
34025-sheep-China                   ......................................------........................................................
34034-sheep-China                   ......................................------........................................................
19141-yak-China                     ......................................------........................................................
19142-yak-China                     ......................................------........................................................
28682-yak-China                     ......................................------........................................................
30241-swamp deer-Nepal              ......................................------........................................................
30238-swamp deer-Nepal              ......................................------........................................................
30243-swamp deer-Nepal              ......................................------........................................................
32563-impala-S.Africa               ......................................------........................................................
32560-buffallo-S.Africa             ......................................------........................................................
31261-human-Turkey                  ......................................------........................................................
37828-human-UK                      ......................................------........................................................
37830-human-UK                      ......................................------........................................................
37827-human-UK                      ......................................------........................................................
31570-sheep-Spain                   ......................................------........................................................
37820-human-UK                      ......................................------........................................................
37811-sheep-UK                      ......................................------........................................................
37809-sheep-UK                      ......................................------........................................................
37813-sheep-UK                      ......................................------........................................................
37815-sheep-UK                      ......................................------........................................................
37808-equine-UK                     ......................................------........................................................
36629-sheep-Spain                   ......................................------........................................................
36628-sheep-Spain                   ......................................------........................................................
18165-sheep-Peru                    ......................................------........................................................
37821-human-UK                      ......................................------........................................................
37818-human-UK                      ......................................------........................................................
37825-human-UK                      ......................................------........................................................
37829-human-UK                      ......................................------........................................................
16421-human-Peru                    ......................................------........................................................
9870-human-Canada                   ......................................------........................................................
19716-alpaca-Peru                   ......................................------........................................................
35147-goat-Algeria                  ............A.........................------........................................................
18868-chipmunk-USA                  AA.A..C..C..A...........T.C.....A.....------.G..T...G.....AA......G................CT.........C.....
33456-sifaka-USA                    AA.A..C..C..A...........T.C.....A.....------.G..T...G.....AA......G................CT.........C.....
```

```
37822-human-UK                  AA.A..C..C..A...........T.C.....A.....------.G..T...G.....AA......G................CT.........C.....
37824-human-UK                  AA.A..C..C..A...........T.C.....A.....------.G..T...G.....AA......G................CT.........C.....
37819-human-UK                  AA.A..C..C..A...........T.C.....A.....------.G..T...G.....AA......G................CT.........C.....
37202-human-USA                 AA.A..C..C..A...........T.C.....A.....------.G..T...G.....AA......G................CT.........C.....
37192-human-USA                 AA.A..C..C..A...........T.C.....A.....------.G..T...G.....AA......G................CT.........C.....
37206-human-USA                 AA.A..C..C..A...........T.C.....A.....------.G..T...G.....AA......G................CT.........C.....
37191-human-USA                 AA.A..C..C..A...........T.C.....A.....------.G..T...G.....AA......G................CT.........C.....
34141-human-USA                 AA.A..C..C..A...........T.C.....A.....------.G..T...G.....AA......G................CT.........C.....
37215-human-USA                 AA.A..C..C..A...........T.C.....A.....------.G..T...G.....AA......G................CT.........C.....
37201-human-USA                 AA.A..C..C..A...........T.C.....A.....------.G..T...G.....AA......G................CT.........C.....
37212-human-USA                 AA.A..C..C..A...........T.C.....A.....------.G..T...G.....AA......G................CT.........C.....
29355-human-USA                 AA.A..C..C..A...........T.C.....A.....------.G..T...G.....AA......G................CT.........C.....
8512-storm-water-USA            AA.A..C..C..A...........T.C.....A.....------.G..T...G.....AA......G................CT.........C.....
8513-storm-water-USA            AA.A..C..C..A...........T.C.....A.....------.G..T...G.....AA......G................CT.........C.....
5397-storm-water-USA            AA.A..C..C..A...........T.C.....A.....------.G..T...G.....AA......G................CT.........C.....
6140-storm-water-USA            AA.A..C..C..A...........T.C.....A.....------.G..T...G.....AA......G................CT.........C.....
8509-storm-water-USA            AA.A..C..C..A...........T.C.....A.....------.G..T...G.....AA......G................CT.........C.....
8469-storm-water-USA            AA.A..C..C..A...........T.C.....A.....------.G..T...G.....AA......G................CT.........C.....
6141-storm-water-USA            AA.A..C..C..A...........T.C.....A.....------.G..T...G.....AA......G................CT.........C.....
8062-storm-water-USA            AA.A..C..C..A...........T.C.....A.....------.G..T...G.....AA......G................CT.........C.....
6143-storm-water-USA            AA.A..C..C..A...........T.C.....A.....------.G..T...G.....AA......G................CT.........C.....
6144-storm-water-USA            AA.A..C..C..A...........T.C.....A.....------.G..T...G.....AA......G................CT.........C.....
8514-storm-water-USA            AA.A..C..C..A...........T.C.....A.....------.G..T...G.....AA......G................CT.........C.....
8510-storm-water-USA            AA.A..C..C..A...........T.C.....A.....------.G..T...G.....AA......G................CT.........C.....
6317-storm-water-USA            AA.A..C..C..A...........T.C.....A.....------.G..T...G.....AA......G................CT.........C.....
7370-storm-water-USA            AA.A..C..C..A...........T.C.....A.....------.G..T...G.....AA......G................CT.........C.....
7163-storm-water-USA            AA.A..C..C..A...........T.C.....A.....------.G..T...G.....AA......G................CT.........C.....
15529-river-water-USA           AA.A..C..C..A...........T.C.....A.....------.G..T...G.....AA......G................CT.........C.....
14874-river-water-USA           AA.A..C..C..A...........T.C.....A.....------.G..T...G.....AA......G................CT.........C.....
18371-eastern gray squirrel-US  AA.A..C..C..A........C..TAC.....C.....------.G..T.........A........................CT...........T...
18370-eastern gray squirrel-US  AA.A..C..C..A........C..TAC.....C.....------.G..T.........A........................CT...........T...
14886-eastern gray squirrel-US  AA.A..C..C..A........C..TAC.....C.....------.G..T.........A........................CT...........T...
14223-beaver-USA                AA.A..C..C..A........C..TAC.....C.....------.G..T.........A........................CT...........T...
13266-red squirrel-USA          AA.A..C..C..A........C..TAC.....C.....------.G..T.........A........................CT...........T...
37200-human-USA                 AA.A..C..C..A........C..TAC.....C.....------.G..T.........A........................CT...........T...
37203-human-USA                 AA.A..C..C..A........C..TAC.....C.....------.G..T.........A........................CT...........T...
37204-human-USA                 AA.A..C..C..A........C..TAC.....C.....------.G..T.........A........................CT...........T...
37197-human-USA                 AA.A..C..C..A........C..TAC.....C.....------.G..T.........A........................CT...........T...
37217-human-USA                 AA.A..C..C..A........C..TAC.....C.....------.G..T.........A........................CT...........T...
37193-human-USA                 AA.A..C..C..A........C..TAC.....C.....------.G..T.........A........................CT...........T...
29361-human-USA                 AA.A..C..C..A........C..TAC.....C.....------.G..T.........A........................CT...........T...
8774-human-USA                  AA.A..C..C..A........C..TAC.....C.....------.G..T.........A........................CT...........T...
37196-human-USA                 AA.A..C..C..A........C..TAC.....C.....------.G..T.........A........................CT...........T...
37208-human-USA                 AA.A..C..C..A........C..TAC.....C.....------.G..T.........A........................CT...........T...
37216-human-USA                 AA.A..C..C..A........C..TAC.....C.....------.G..T.........A........................CT...........T...
37209-human-USA                 AA.A..C..C..A........C..TAC.....C.....------.G..T.........A........................CT...........T...
37826-human-UK                  AA.A..C..C..A........C..TAC.....C.....------.G..T.........A........................CT...........T...
37833-human-UK                  AA.A..C..C..A........C..TAC.....C.....------.G..T.........A........................CT...........T...
22508-river-water-USA           AA.A..C..C..A........C..TAC.....C.....------.G..T.........A........................CT...........T...
13264-woodchuck-USA             AA.A..C..C..A........C..TAC.....C.....------.G..T.........A........................CT...........T...
12963-raccoon-USA               AA.A..C..C..A........C..T.C.....C.....------.G..T.........A........................CT...........T...
36641-porcupine-USA             AA.A..C..C..A........C..TAC.....C.....------.G..T.........AA.......................CT...........T...
36642-porcupine-USA             AA.A..C..C..A........C..TAC.....C.....------.G..T.........AA.......................CT...........T...
36638-porcupine via goat-USA    AA.A..C..C..A........C..TAC.....C.....------.G..T.........AA.......................CT...........T...
36639-porcupine via goat-USA    AA.A..C..C..A........C..TAC.....C.....------.G..T.........AA.......................CT...........T...
36640-porcupine via goat-USA    AA.A..C..C..A........C..TAC.....C.....------.G..T.........AA.......................CT...........T...
17654-human-USA                 AA.A..C..C..A........C..TAC.....C.....------.G..T.........AA.......................CT...........T...
17577-human-USA                 AA.A..C..C..A........C..TAC.....C.....------.G..T.........AA.......................CT...........T...
37211-human-USA                 AA.A..C..C..A........C..TAC.....C.....------.G..T.........AA.......................CT...........T...
17574-human-USA                 AA.A..C..C..A........C..TAC.....C.....------.G..T.........AA.......................CT...........T...
37640-field mouse-Slovak Repub  ...A........A...........CAC..C.GCTC...------.GA.T...G.T...A......................A.CT.....G.........
37642-field mouse-Slovak Repub  ...A........A...........CAC..C.GCTC...------.GA.T...G.T...A......................A.CT.....G.........
37644-field mouse-Slovak Repub  ...A........A...........CAC..C.GCTC...------.GA.T...G.T...A......................A.CT.....G.........
37646-field mouse-Slovak Repub  A.AA..C..C..A...........T.C.....C.....------.G............A.T...........A..C.....A.CT.....T...C.G...
AF114166-C. parvum-gp60 gene    TGAA.....AT.T..A..C.T....A.G..TGAT..T.CAGTTA..A...A.G.T..C..T.....T...AG......C..TGCTA.....AGT.GT...
Clustal Consensus                   ** **  * ** ** *  **    *      * *      *  * * * * **    ***** ***   ** ** **     ****       ***

                                        810       820       830       840       850       860       870       880       890       900
                                ....|....|....|....|....|....|....|....|....|....|....|....|....|....|....|....|....|....|....|....|
36625-sheep-Brazil              ACCAC---AGCTACAACAACACCAGCAGCTAGGTTACT-TGCAGAAGGTGGCACT--------GCTACAGAAGCCGTCGCAATGACTGATCTATACACCT
36624-sheep-Brazil              .....---..............................-................--------.....................................
36623-sheep-Brazil              .....---..............................-................--------.....................................
36630-sheep-Spain               .....---..............................-................--------.....................................
36631-sheep-Spain               .....---..............................-................--------.....................................
36633-sheep-Spain               .....---..............................-................--------.....................................
36627-sheep-Spain               .....---..............................-................--------.....................................
36632-sheep-Spain               .....---..............................-................--------.....................................
36626-sheep-Spain               .....---..............................-................--------.....................................
36634-sheep-USA                 .....---..............................-................--------.....................................
36637-sheep-USA                 .....---..............................-................--------.....................................
37766-sheep-USA                 .....---..............................-................--------.....................................
36636-sheep-USA                 .....---..............................-................--------.....................................
37769-sheep-USA                 .....---..............................-................--------.....................................
37767-sheep-USA                 .....---..............................-................--------.....................................
37768-sheep-USA                 .....---..............................-................--------.....................................
37774-sheep-USA                 .....---..............................-................--------.....................................
37777-sheep-USA                 .....---..............................-................--------.....................................
37776-sheep-USA                 .....---..............................-................--------.....................................
37775-sheep-USA                 .....---..............................-................--------.....................................
37772-sheep-USA                 .....---..............................-................--------.....................................
37773-sheep-USA                 .....---..............................-................--------.....................................
37771-sheep-USA                 .....---..............................-................--------.....................................
6478-blesbok-Czech Republic     .....---..............................-................--------.....................................
6482-nyala-Czech Republic       .....---..............................-................--------.....................................
34023-sheep-China               .....---..............................-................--------.....................................
36737-sheep-China               .....---..............................-................--------.....................................
34022-sheep-China               .....---..............................-................--------.....................................
38161-sheep-Australia           .....---..............................-................--------.....................................
38157-sheep-Australia           .....---..............................-................--------.....................................
38156-sheep-Australia           .....---..............................-................--------.....................................
38154-sheep-Australia           .....---..............................-................--------.....................................
38153-sheep-Australia           .....---..............................-................--------.....................................
38152-sheep-Australia           .....---..............................-................--------.....................................
```

```
34049-sheep-China                 .....---..............................-................--------.....................................
37635-sheep-Turkey                .....---..............................-................--------.....................................
34043-sheep-China                 .....---..............................-................--------.....................................
34047-sheep-China                 .....---..............................-................--------.....................................
34055-sheep-China                 .....---..............................-................--------.....................................
34011-sheep-China                 .....---..............................-................--------.....................................
34038-sheep-China                 .....---..............................-................--------.....................................
34044-sheep-China                 .....---..............................-................--------.....................................
34032-sheep-China                 .....---..............................-................--------.....................................
34060-sheep-China                 .....---..............................-................--------.....................................
34048-sheep-China                 .....---..............................-................--------.....................................
36745-sheep-China                 .....---..............................-................--------.....................................
36785-sheep-China                 .....---..............................-................--------.....................................
34025-sheep-China                 .....---..............................-................--------.....................................
34034-sheep-China                 .....---..............................-................--------.....................................
19141-yak-China                   .....---..............................-................--------.....................................
19142-yak-China                   .....---..............................-................--------.....................................
28682-yak-China                   .....---..............................-................--------.....................................
30241-swamp deer-Nepal            .....---..............................-................--------.....................................
30238-swamp deer-Nepal            .....---..............................-................--------.....................................
30243-swamp deer-Nepal            .....---..............................-................--------.....................................
32563-impala-S.Africa             .....---..............................-................--------.....................................
32560-buffallo-S.Africa           .....---..............................-................--------.....................................
31261-human-Turkey                .....---..............................-................--------.....................................
37828-human-UK                    .....---..............................-................--------.....................................
37830-human-UK                    .....---..............................-................--------.....................................
37827-human-UK                    .....---..............................-................--------.....................................
31570-sheep-Spain                 .....---..............................-................--------.....................................
37820-human-UK                    .....---..............................-................--------.....................................
37811-sheep-UK                    .....---..............................-................--------.....................................
37809-sheep-UK                    .....---..............................-................--------.....................................
37813-sheep-UK                    .....---..............................-................--------.....................................
37815-sheep-UK                    .....---..............................-................--------.....................................
37808-equine-UK                   .....---..............................-................--------.....................................
36629-sheep-Spain                 .....---..............................-................--------.....................................
36628-sheep-Spain                 .....---..............................-................--------.....................................
18165-sheep-Peru                  .....---..............................-................--------.....................................
37821-human-UK                    .....---..............................-................--------.....................................
37818-human-UK                    .....---..............................-................--------.....................................
37825-human-UK                    .....---..............................-................--------.....................................
37829-human-UK                    .....---..............................-................--------.....................................
16421-human-Peru                  .....---..............................-................--------.....................................
9870-human-Canada                 .....---..............................-................--------.....................................
19716-alpaca-Peru                 .....---..............G...............-................--------.....................................
35147-goat-Algeria                .....---..............................-................--------.....................................
18868-chipmunk-USA                .....---..........G...................-........---AT...--------.T.............A...........T.........
33456-sifaka-USA                  .....---..........G...................-........---AT...--------.T.............A...........T.........
37822-human-UK                    .....---..........G...................-........---AT...--------.T.............A...........T.........
37824-human-UK                    .....---..........G...................-........---AT...--------.T.............A...........T.........
37819-human-UK                    .....---..........G...................-........---AT...--------.T.............A...........T.........
37202-human-USA                   .....---..........G...................-........---AT...--------.T.............A...........T.........
37192-human-USA                   .....---..........G...................-........---AT...--------.T.............A...........T.........
37206-human-USA                   .....---..........G...................-........---AT...--------.T.............A...........T.........
37191-human-USA                   .....---..........G...................-........---AT...--------.T.............A...........T.........
34141-human-USA                   .....---..........G...................-........---AT...--------.T.............A...........T.........
37215-human-USA                   .....---..........G...................-........---AT...--------.T.............A...........T.........
37201-human-USA                   .....---..........G...................-........---AT...--------.T.............A...........T.........
37212-human-USA                   .....---..........G...................-........---AT...--------.T.............A...........T.........
29355-human-USA                   .....---..........G...................-........---AT...--------.T.............A...........T.........
8512-storm-water-USA              .....---..........G...................-........---AT...--------.T.............A...........T.........
8513-storm-water-USA              .....---..........G...................-........---AT...--------.T.............A...........T.........
5397-storm-water-USA              .....---..........G...................-........---AT...--------.T.............A...........T.........
6140-storm-water-USA              .....---..........G...................-........---AT...--------.T.............A...........T.........
8509-storm-water-USA              .....---..........G...................-........---AT...--------.T.............A...........T.........
8469-storm-water-USA              .....---..........G...................-........---AT...--------.T.............A...........T.........
6141-storm-water-USA              .....---..........G...................-........---AT...--------.T.............A...........T.........
8062-storm-water-USA              .....---..........G...................-........---AT...--------.T.............A...........T.........
6143-storm-water-USA              .....---..........G...................-........---AT...--------.T.............A...........T.........
6144-storm-water-USA              .....---..........G...................-........---AT...--------.T.............A...........T.........
8514-storm-water-USA              .....---..........G...................-........---AT...--------.T.............A...........T.........
8510-storm-water-USA              .....---..........G...................-........---AT...--------.T.............A...........T.........
6317-storm-water-USA              .....---..........G...................-........---AT...--------.T.............A...........T.........
7370-storm-water-USA              .....---..........G...................-........---AT...--------.T.............A...........T.........
7163-storm-water-USA              .....---..........G...................-........---AT...--------.T.............A...........T.........
15529-river-water-USA             .....---..........G...................-........---AT...--------.T.............A...........T.........
14874-river-water-USA             .....---..........G...................-........---AT...--------.T.............A...........T.........
18371-eastern gray squirrel-US    .....CTC..G.T.TG........T.............-C........C.A....--------.....................................
18370-eastern gray squirrel-US    .....CTC..G.T.TG........T.............-C........C.A....--------.....................................
14886-eastern gray squirrel-US    .....CTC..G.T.TG........T.............-C........C.A....--------.....................................
14223-beaver-USA                  .....CTC..G.T.TG........T.............-C........C.A....--------.....................................
13266-red squirrel-USA            .....CTC..G.T.TG........T.............-C........C.A....--------.....................................
37200-human-USA                   .....CTC..G.T.TG........T.............-C........C.A....--------.....................................
37203-human-USA                   .....CTC..G.T.TG........T.............-C........C.A....--------.....................................
37204-human-USA                   .....CTC..G.T.TG........T.............-C........C.A....--------.....................................
37197-human-USA                   .....CTC..G.T.TG........T.............-C........C.A....--------.....................................
37217-human-USA                   .....CTC..G.T.TG........T.............-C........C.A....--------.....................................
37193-human-USA                   .....CTC..G.T.TG........T.............-C........C.A....--------.....................................
29361-human-USA                   .....CTC..G.T.TG........T.............-C........C.A....--------.....................................
8774-human-USA                    .....CTC..G.T.TG........T.............-C........C.A....--------.....................................
37196-human-USA                   .....CTC..G.T.TG........T.............-C........C.A....--------.....................................
37208-human-USA                   .....CTC..G.T.TG........T.............-C........C.A....--------.....................................
37216-human-USA                   .....CTC..G.T.TG........T.............-C........C.A....--------.....................................
37209-human-USA                   .....CTC..G.T.TG........T.............-C........C.A....--------.....................................
37826-human-UK                    .....CTC..G.T.TG........T.............-C........C.A....--------.....................................
37833-human-UK                    .....CTC..G.T.TG........T.............-C........C.A....--------.....................................
22508-river-water-USA             .....CTC..G.T.TG........T.............-C........C.A....--------.....................................
13264-woodchuck-USA               .....CTC..G.T.TG........T.............-C........C.A....--------.....................................
12963-raccoon-USA                 .....CTC..G.T.TG........T.............-C........C.A....--------.....................................
36641-porcupine-USA               .....CTC..G.T.TG..GA....T.............-C........C.A....--------.....................................
36642-porcupine-USA               .....CTC..G.T.TG..GA....T.............-C........C.A....--------.....................................
36638-porcupine via goat-USA      .....CTC..G.T.TG..GA....T.............-C........C.A....--------.....................................
36639-porcupine via goat-USA      .....CTC..G.T.TG..GA....T.............-C........C.A....--------.....................................
36640-porcupine via goat-USA      .....CTC..G.T.TG..GA....T.............-C........C.A....--------.....................................
```

```
17654-human-USA                 .....CTC..G.T.TG..GA....T.............-C........C.A....--------.....................................
17577-human-USA                 .....CTC..G.T.TG..GA....T.............-C........C.A....--------.....................................
37211-human-USA                 .....CTC..G.T.TG..GA....T.............-C........C.A....--------.....................................
17574-human-USA                 .....CTC..G.T.TG..GA....T.............-C........C.A....--------.....................................
37640-field mouse-Slovak Repub  ..TGG---.T.A......---------.T....C....-.CA...TA...--------------.......G..T...A...........G.T....GT.
37642-field mouse-Slovak Repub  ..TGG---.T.A......---------.T....C....-.CA...TA...--------------.......G..T...A...........G.T....GT.
37644-field mouse-Slovak Repub  ..TGG---.T.A......---------.T....C....-.CA...TA...--------------.......G..T...A...........G.T....GT.
37646-field mouse-Slovak Repub  ..GGG---.T.A......---------.T...A..G..-.CA...T...A--------------.......G..T.................C.....T.
AF114166-C. parvum-gp60 gene    ..TGAAAAT.GCGG.T.TG.GGGTCAG...TCA.C.AGA.CA.....A.CA.T..CAGAGGAAA.C.GT.....T.CT....CCGTC...T.G.TTG...
Clustal Consensus               **              *          * *            ***                     *  ** ** *   ***     *** * *     *

                                        910       920       930       940       950       960       970       980       990      1000
                                ....|....|....|....|....|....|....|....|....|....|....|....|....|....|....|....|....|....|....|....|
36625-sheep-Brazil              TCACCTTAAAGGGTGGTAAAGCCATTAGTGTAGGTGTGCCTGCTGTGGACGATTCAACTAAAAGAGACAAATACAGTTTATCCGCTGATAGCCAAACATT
36624-sheep-Brazil              ....................................................................................................
36623-sheep-Brazil              ....................................................................................................
36630-sheep-Spain               ....................................................................................................
36631-sheep-Spain               ....................................................................................................
36633-sheep-Spain               ....................................................................................................
36627-sheep-Spain               ....................................................................................................
36632-sheep-Spain               ....................................................................................................
36626-sheep-Spain               ....................................................................................................
36634-sheep-USA                 ....................................................................................................
36637-sheep-USA                 ....................................................................................................
37766-sheep-USA                 ....................................................................................................
36636-sheep-USA                 ....................................................................................................
37769-sheep-USA                 ....................................................................................................
37767-sheep-USA                 ....................................................................................................
37768-sheep-USA                 ....................................................................................................
37774-sheep-USA                 ....................................................................................................
37777-sheep-USA                 ....................................................................................................
37776-sheep-USA                 ....................................................................................................
37775-sheep-USA                 ....................................................................................................
37772-sheep-USA                 ....................................................................................................
37773-sheep-USA                 ....................................................................................................
37771-sheep-USA                 ....................................................................................................
6478-blesbok-Czech Republic     ....................................................................................................
6482-nyala-Czech Republic       ....................................................................................................
34023-sheep-China               ....................................................................................................
36737-sheep-China               ....................................................................................................
34022-sheep-China               ....................................................................................................
38161-sheep-Australia           ....................................................................................................
38157-sheep-Australia           ....................................................................................................
38156-sheep-Australia           ....................................................................................................
38154-sheep-Australia           ....................................................................................................
38153-sheep-Australia           ....................................................................................................
38152-sheep-Australia           ....................................................................................................
34049-sheep-China               ....................................................................................................
37635-sheep-Turkey              ....................................................................................................
34043-sheep-China               ....................................................................................................
34047-sheep-China               ....................................................................................................
34055-sheep-China               ....................................................................................................
34011-sheep-China               ....................................................................................................
34038-sheep-China               ....................................................................................................
34044-sheep-China               ....................................................................................................
34032-sheep-China               ....................................................................................................
34060-sheep-China               ....................................................................................................
34048-sheep-China               ....................................................................................................
36745-sheep-China               ....................................................................................................
36785-sheep-China               ....................................................................................................
34025-sheep-China               ....................................................................................................
34034-sheep-China               ....................................................................................................
19141-yak-China                 ....................................................................................................
19142-yak-China                 ....................................................................................................
28682-yak-China                 ....................................................................................................
30241-swamp deer-Nepal          ....................................................................................................
30238-swamp deer-Nepal          ....................................................................................................
30243-swamp deer-Nepal          ....................................................................................................
32563-impala-S.Africa           ....................................................................................................
32560-buffallo-S.Africa         ....................................................................................................
31261-human-Turkey              ....................................................................................................
37828-human-UK                  ....................................................................................................
37830-human-UK                  .................................-..................................................................
37827-human-UK                  ....................................................................................................
31570-sheep-Spain               ....................................................................................................
37820-human-UK                  ....................................................................................................
37811-sheep-UK                  ....................................................................................................
37809-sheep-UK                  ....................................................................................................
37813-sheep-UK                  ....................................................................................................
37815-sheep-UK                  ....................................................................................................
37808-equine-UK                 ....................................................................................................
36629-sheep-Spain               ....................................................................................................
36628-sheep-Spain               ....................................................................................................
18165-sheep-Peru                ....................................................................................................
37821-human-UK                  ....................................................................................................
37818-human-UK                  ....................................................................................................
37825-human-UK                  ....................................................................................................
37829-human-UK                  ....................................................................................................
16421-human-Peru                ....................................................................................................
9870-human-Canada               ....................................................................................................
19716-alpaca-Peru               ....................................................................................................
35147-goat-Algeria              ....................................................................................................
18868-chipmunk-USA              ...................G........C.....C.....A...AACC.A..CGA..G...G............................A...GGT...
33456-sifaka-USA                ...................G........C.....C.....A...AACC.A..CGA..G...G............................A...GGT...
37822-human-UK                  ...................G........C.....C.....A...AACC.A..CGA..G...G............................A...GGT...
37824-human-UK                  ...................G........C.....C.....A...AACC.A..CGA..G...G............................A...GGT...
37819-human-UK                  ...................G........C.....C.....A...AACC.A..CGA..G...G............................A...GGT...
37202-human-USA                 ...................G........C.....C.....A...AACC.A..CGA..G...G............................A...GGT...
37192-human-USA                 ...................G........C.....C.....A...AACC.A..CGA..G...G............................A...GGT...
37206-human-USA                 ...................G........C.....C.....A...AACC.A..CGA..G...G............................A...GGT...
37191-human-USA                 ...................G........C.....C.....A...AACC.A..CGA..G...G............................A...GGT...
34141-human-USA                 ...................G........C.....C.....A...AACC.A..CGA..G...G............................A...GGT...
37215-human-USA                 ...................G........C.....C.....A...AACC.A..CGA..G...G............................A...GGT...
```

```
37201-human-USA                     ...................G........C.....C.....A...AACC.A..CGA..G...G............................A...GGT...
37212-human-USA                     ...................G........C.....C.....A...AACC.A..CGA..G...G............................A...GGT...
29355-human-USA                     ...................G........C.....C.....A...AACC.A..CGA..G...G............................A...GGT...
8512-storm-water-USA                ...................G........C.....C.....A...AACC.A..CGA..G...G............................A...GGT...
8513-storm-water-USA                ...................G........C.....C.....A...AACC.A..CGA..G...G............................A...GGT...
5397-storm-water-USA                ...................G........C.....C.....A...AACC.A..CGA..G...G............................A...GGT...
6140-storm-water-USA                ...................G........C.....C.....A...AACC.A..CGA..G...G............................A...GGT...
8509-storm-water-USA                ...................G........C.....C.....A...AACC.A..CGA..G...G............................A...GGT...
8469-storm-water-USA                ...................G........C.....C.....A...AACC.A..CGA..G...G............................A...GGT...
6141-storm-water-USA                ...................G........C.....C.....A...AACC.A..CGA..G...G............................A...GGT...
8062-storm-water-USA                ...................G........C.....C.....A...AACC.A..CGA..G...G............................A...GGT...
6143-storm-water-USA                ...................G........C.....C.....A...AACC.A..CGA..G...G............................A...GGT...
6144-storm-water-USA                ...................G........C.....C.....A...AACC.A..CGA..G...G............................A...GGT...
8514-storm-water-USA                ...................G........C.....C.....A...AACC.A..CGA..G...G............................A...GGT...
8510-storm-water-USA                ...................G........C.....C.....A...AACC.A..CGA..G...G............................A...GGT...
6317-storm-water-USA                ...................G........C.....C.....A...AACC.A..CGA..G...G............................A...GGT...
7370-storm-water-USA                ...................G........C.....C.....A...AACC.A..CGA..G...G............................A...GGT...
7163-storm-water-USA                ...................G........C.....C.....A...AACC.A..CGA..G...G............................A...GGT...
15529-river-water-USA               ...................G........C.....C.....A...AACC.A..CGA..G...G............................A...GGT...
14874-river-water-USA               ...................G........C.....C.....A...AACC.A..CGA..G...G............................A...GGT...
18371-eastern gray squirrel-US      ...................G.................A..A.T.AAC..A..CC.......G............................A....GT...
18370-eastern gray squirrel-US      ...................G.................A..A.T.AAC..A..CC.......G............................A....GT...
14886-eastern gray squirrel-US      ...................G.................A..A.T.AAC..A..CC.......G............................A....GT...
14223-beaver-USA                    ...................G.................A..A.T.AAC..A..CC.......G............................A....GT...
13266-red squirrel-USA              ...................G.................A..A.T.AAC..A..CC.......G............................A....GT...
37200-human-USA                     ...................G.................A..A.T.AAC..A..CC.......G............................A....GT...
37203-human-USA                     ...................G.................A..A.T.AAC..A..CC.......G............................A....GT...
37204-human-USA                     ...................G.................A..A.T.AAC..A..CC.......G............................A....GT...
37197-human-USA                     ...................G.................A..A.T.AAC..A..CC.......G............................A....GT...
37217-human-USA                     ...................G.................A..A.T.AAC..A..CC.......G............................A....GT...
37193-human-USA                     ...................G.................A..A.T.AAC..A..CC.......G............................A....GT...
29361-human-USA                     ...................G.................A..A.T.AAC..A..CC.......G............................A....GT...
8774-human-USA                      ...................G.................A..A.T.AAC..A..CC.......G............................A....GT...
37196-human-USA                     ...................G.................A..A.T.AAC..A..CC.......G............................A....GT...
37208-human-USA                     ...................G.................A..A.T.AAC..A..CC.......G............................A....GT...
37216-human-USA                     ...................G.................A..A.T.AAC..A..CC.......G............................A....GT...
37209-human-USA                     ...................G.................A..A.T.AAC..A..CC.......G............................A....GT...
37826-human-UK                      ...................G.................A..A.T.AAC..A..CC.......G............................A....GT...
37833-human-UK                      ...................G.................A..A.T.AAC..A..CC.......G............................A....GT...
22508-river-water-USA               ...................G.................A..A.T.AAC..A..CC.......G............................A....GT...
13264-woodchuck-USA                 ...................G.................A..A.T.AAC..A..CC.......G............................A....GT...
12963-raccoon-USA                   ...................G.................A..A.T.AAC..A..CC.......G............................A....GT...
36641-porcupine-USA                 ...................G.................A..A.T.AAC..A..CC.......G..............C..G..T.................
36642-porcupine-USA                 ...................G.................A..A.T.AAC..A..CC.......G..............C..G..T.................
36638-porcupine via goat-USA        ...................G.................A..A.T.AAC..A..CC.......G..............C..G..T.................
36639-porcupine via goat-USA        ...................G.................A..A.T.AAC..A..CC.......G..............C..G..T.................
36640-porcupine via goat-USA        ...................G.................A..A.T.AAC..A..CC.......G..............C..G..T.................
17654-human-USA                     ...................G.................A..A.T.AAC..A..CC.......G..............C..G..T.................
17577-human-USA                     ...................G.................A..A.T.AAC..A..CC.......G..............C..G..T.................
37211-human-USA                     ...................G.................A..A.T.AAC..A..CC.......G..............C..G..T.................
17574-human-USA                     ...................G.................A..A.T.AAC..A..CC.......G..............C..G..T.................
37640-field mouse-Slovak Repub      .T.....G..A........GCT.........G.....A..A....ATTC....G....C..G........G....C....AGT..C..............
37642-field mouse-Slovak Repub      .T.....G..A........GCT.........G.....A..A....ATTC....G....C..G........G....C....AGT..C..............
37644-field mouse-Slovak Repub      .T.....G..A........GCT.........G.....A..A....ATTC....G....C..G........G....C....AGT..C..............
37646-field mouse-Slovak Repub      ....T...........C..G..T.................A....AAT.A...C.G...TTG........G........GAGT..C....A....G....
AF114166-C. parvum-gp60 gene        .T...C.TG.T.........AGA...GAA..G.C...A..AAAC..C..A...G..T.............G........GGTT..A..CGATA..C.T..
Clustal Consensus                   * **  *  * ***** **    ***   ** *  ** **          **          ******** ****  **    ** **     *    **

                                             1010      1020      1030      1040      1050      1060      1070
                                    ....|....|....|....|....|....|....|....|....|....|....|....|....|....|..
36625-sheep-Brazil                  CTACACCGGTACTGCTACCAATAGTGGTACTACTAATGGAATCTTCAAATTGAACAATGATGGTGATTTAGT
36624-sheep-Brazil                  ........................................................................
36623-sheep-Brazil                  ........................................................................
36630-sheep-Spain                   ........................................................................
36631-sheep-Spain                   ........................................................................
36633-sheep-Spain                   ........................................................................
36627-sheep-Spain                   ........................................................................
36632-sheep-Spain                   ........................................................................
36626-sheep-Spain                   ........................................................................
36634-sheep-USA                     ........................................................................
36637-sheep-USA                     ........................................................................
37766-sheep-USA                     ........................................................................
36636-sheep-USA                     ........................................................................
37769-sheep-USA                     ........................................................................
37767-sheep-USA                     ........................................................................
37768-sheep-USA                     ........................................................................
37774-sheep-USA                     ........................................................................
37777-sheep-USA                     ........................................................................
37776-sheep-USA                     ........................................................................
37775-sheep-USA                     ........................................................................
37772-sheep-USA                     ........................................................................
37773-sheep-USA                     ........................................................................
37771-sheep-USA                     ........................................................................
6478-blesbok-Czech Republic         ........................................................................
6482-nyala-Czech Republic           ........................................................................
34023-sheep-China                   ........................................................................
36737-sheep-China                   ........................................................................
34022-sheep-China                   ........................................................................
38161-sheep-Australia               ........................................................................
38157-sheep-Australia               ........................................................................
38156-sheep-Australia               ........................................................................
38154-sheep-Australia               ........................................................................
38153-sheep-Australia               ........................................................................
38152-sheep-Australia               ........................................................................
34049-sheep-China                   ........................................................................
37635-sheep-Turkey                  ........................................................................
34043-sheep-China                   ........................................................................
34047-sheep-China                   ........................................................................
34055-sheep-China                   ........................................................................
34011-sheep-China                   ........................................................................
34038-sheep-China                   ........................................................................
34044-sheep-China                   ........................................................................
34032-sheep-China                   ........................................................................
```

```
34060-sheep-China                  ........................................................................
34048-sheep-China                  ........................................................................
36745-sheep-China                  ........................................................................
36785-sheep-China                  ........................................................................
34025-sheep-China                  ........................................................................
34034-sheep-China                  ........................................................................
19141-yak-China                    ........................................................................
19142-yak-China                    ........................................................................
28682-yak-China                    ........................................................................
30241-swamp deer-Nepal             ........................................................................
30238-swamp deer-Nepal             ........................................................................
30243-swamp deer-Nepal             ........................................................................
32563-impala-S.Africa              ........................................................................
32560-buffallo-S.Africa            ........................................................................
31261-human-Turkey                 ........................................................................
37828-human-UK                     ........................................................................
37830-human-UK                     ........................................................................
37827-human-UK                     ........................................................................
31570-sheep-Spain                  ........................................................................
37820-human-UK                     ........................................................................
37811-sheep-UK                     ........................................................................
37809-sheep-UK                     ........................................................................
37813-sheep-UK                     ........................................................................
37815-sheep-UK                     ........................................................................
37808-equine-UK                    ........................................................................
36629-sheep-Spain                  ........................................................................
36628-sheep-Spain                  ........................................................................
18165-sheep-Peru                   ........................................................................
37821-human-UK                     ........................................................................
37818-human-UK                     ........................................................................
37825-human-UK                     ........................................................................
37829-human-UK                     ........................................................................
16421-human-Peru                   ........................................................................
9870-human-Canada                  ........................................................................
19716-alpaca-Peru                  ........................................................................
35147-goat-Algeria                 ........................................................................
18868-chipmunk-USA                 ...T..T..........G..........GTC....G......A............G.GA.......C.....
33456-sifaka-USA                   ...T..T..........G..........GTC....G......A............G.GA.......C.....
37822-human-UK                     ...T..T..........G..........GTC....G......A............G.GA.......C.....
37824-human-UK                     ...T..T..........G..........GTC....G......A............G.GA.......C.....
37819-human-UK                     ...T..T..........G..........GTC....G......A............G.GA.......C.....
37202-human-USA                    ...T..T..........G..........GTC....G......A............G.GA.......C.....
37192-human-USA                    ...T..T..........G..........GTC....G......A............G.GA.......C.....
37206-human-USA                    ...T..T..........G..........GTC....G......A............G.GA.......C.....
37191-human-USA                    ...T..T..........G..........GTC....G......A............G.GA.......C.....
34141-human-USA                    ...T..T..........G..........GTC....G......A............G.GA.......C.....
37215-human-USA                    ...T..T..........G..........GTC....G......A............G.GA.......C.....
37201-human-USA                    ...T..T..........G..........GTC....G......A............G.GA.......C.....
37212-human-USA                    ...T..T..........G..........GTC....G......A............G.GA.......C.....
29355-human-USA                    ...T..T..........G..........GTC....G......A............G.GA.......C.....
8512-storm-water-USA               ...T..T..........G..........GTC....G......A............G.GA.......C.....
8513-storm-water-USA               ...T..T..........G..........GTC....G......A............G.GA.......C.....
5397-storm-water-USA               ...T..T..........G..........GTC....G......A............G.GA.......C.....
6140-storm-water-USA               ...T..T..........G..........GTC....G......A............G.GA.......C.....
8509-storm-water-USA               ...T..T..........G..........GTC....G......A............G.GA.......C.....
8469-storm-water-USA               ...T..T..........G..........GTC....G......A............G.GA.......C.....
6141-storm-water-USA               ...T..T..........G..........GTC....G......A............G.GA.......C.....
8062-storm-water-USA               ...T..T..........G..........GTC....G......A............G.GA.......C.....
6143-storm-water-USA               ...T..T..........G..........GTC....G......A............G.GA.......C.....
6144-storm-water-USA               ...T..T..........G..........GTC....G......A............G.GA.......C.....
8514-storm-water-USA               ...T..T..........G..........GTC....G......A............G.GA.......C.....
8510-storm-water-USA               ...T..T..........G..........GTC....G......A............G.GA.......C.....
6317-storm-water-USA               ...T..T..........G..........GTC....G......A............G.GA.......C.....
7370-storm-water-USA               ...T..T..........G..........GTC....G......A............G.GA.......C.....
7163-storm-water-USA               ...T..T..........G..........GTC....G......A............G.GA.......C.....
15529-river-water-USA              ...T..T..........G..........GTC....G......A............G.GA.......C.....
14874-river-water-USA              ...T..T..........G..........GTC....G......A............G.GA.......C.....
18371-eastern gray squirrel-US     ...T.........A...G.G....C.....C....G....C.G............G.G........C.....
18370-eastern gray squirrel-US     ...T.........A...G.G....C.....C....G....C.G............G.G........C.....
14886-eastern gray squirrel-US     ...T.........A...G.G....C.....C....G....C.G............G.G........C.....
14223-beaver-USA                   ...T.........A...G.G....C.....C....G....C.G............G.G........C.....
13266-red squirrel-USA             ...T.........A...G.G....C.....C....G....C.G............G.G........C.....
37200-human-USA                    ...T.........A...G.G....C.....C....G....C.G............G.G........C.....
37203-human-USA                    ...T.........A...G.G....C.....C....G....C.G............G.G........C.....
37204-human-USA                    ...T.........A...G.G....C.....C....G....C.G............G.G........C.....
37197-human-USA                    ...T.........A...G.G....C.....C....G....C.G............G.G........C.....
37217-human-USA                    ...T.........A...G.G....C.....C....G....C.G............G.G........C.....
37193-human-USA                    ...T.........A...G.G....C.....C....G....C.G............G.G........C.....
29361-human-USA                    ...T.........A...G.G....C.....C....G....C.G............G.G........C.....
8774-human-USA                     ...T.........A...G.G....C.....C....G....C.G............G.G........C.....
37196-human-USA                    ...T.........A...G.G....C.....C....G....C.G............G.G........C.....
37208-human-USA                    ...T.........A...G.G....C.....C....G....C.G............G.G........C.....
37216-human-USA                    ...T.........A...G.G....C.....C....G....C.G............G.G........C.....
37209-human-USA                    ...T.........A...G.G....C.....C....G....C.G............G.G........C.....
37826-human-UK                     ...T.........A...G.G....C.....C....G....C.G............G.G........C.....
37833-human-UK                     ...T.........A...G.G....C.....C....G....C.G............G.G........C.....
22508-river-water-USA              ...T.........A...G.G....C.....C....G....C.G............G.G........C.....
13264-woodchuck-USA                ...T.........A...G.G....C.....C....G....C.G............G.G........C.....
12963-raccoon-USA                  ...T.........A...G.G....C.....C....G....C.G............G.G........C.....
36641-porcupine-USA                .......................................................G................
36642-porcupine-USA                .......................................................G................
36638-porcupine via goat-USA       .......................................................G................
36639-porcupine via goat-USA       .......................................................G................
36640-porcupine via goat-USA       .......................................................G................
17654-human-USA                    .......................................................G................
17577-human-USA                    .......................................................G................
37211-human-USA                    .......................................................G................
17574-human-USA                    .......................................................G................
37640-field mouse-Slovak Repub     ......T.........GT...C......GG............A..T.........G....G...........
37642-field mouse-Slovak Repub     ......T.........GT...C......GG............A..T.........G....G...........
37644-field mouse-Slovak Repub     ......T.........GT...C......GG............A..T.........G....G...........
37646-field mouse-Slovak Repub     ......T.........G.T..C......G...........G.G..T.........G..........C.....
AF114166-C. parvum-gp60 gene       ...T.....CG.A------..C..C..C.....C.....TG...A..GG.....TG.GA.C..A..C..G..
```

Clustal Consensus *** ** ** * * ** ** ** * *** * * * ***** * * ** ** ** **

Technical Appendix Figure. Nucleotide alignment of the 60-kDa glycoprotein (gp60) gene of Cryptosporidium ubiquitum. Dots denote nucleotide identity to the sequence of specimen no. 36625, and dashes denote nucleotide deletions.
